# A Synthetic Small Molecule F240B Decreases NLRP3 Inflammasome Activation by Autophagy Induction

**DOI:** 10.3389/fimmu.2020.607564

**Published:** 2020-12-18

**Authors:** Chun-Hsien Wu, Chin Heng Gan, Lan-Hui Li, Jen-Che Chang, Shin-Tai Chen, Mridula P. Menon, Shu-Meng Cheng, Shih-Ping Yang, Chen-Lung Ho, Oleg V. Chernikov, Chi-Hung Lin, Yulin Lam, Kuo-Feng Hua

**Affiliations:** ^1^ Division of Cardiology, Department of Internal Medicine, Tri-Service General Hospital, National Defense Medical Center, Taipei, Taiwan; ^2^ Institute of Microbiology and Immunology, National Yang-Ming University, Taipei, Taiwan; ^3^ Department of Chemistry, National University of Singapore, Singapore, Singapore; ^4^ Department of Laboratory Medicine, Linsen, Chinese Medicine and Kunming Branch, Taipei City Hospital, Taipei, Taiwan; ^5^ Department of Pathology, Tri-Service General Hospital, National Defense Medical Center, Taipei, Taiwan; ^6^ Department of Biotechnology and Animal Science, National Ilan University, Ilan, Taiwan; ^7^ Division of Wood Cellulose, Taiwan Forestry Research Institute, Taipei, Taiwan; ^8^ G.B. Elyakov Pacific Institute of Bioorganic Chemistry FEB RAS, Vladivostok, Russia; ^9^ Department of Biological Science & Technology, National Chiao Tung University, Hsinchu, Taiwan; ^10^ Department of Medical Research, China Medical University Hospital, China Medical University, Taichung, Taiwan

**Keywords:** NLRP3 inflammasome, conjugated polyenes, autophagy, mitochondria, peritonitis

## Abstract

Conjugated polyenes are a class of widely occurring natural products with various biological functions. We previously identified 4-hydroxy auxarconjugatin B (4-HAB) as anti‐inflammatory agent with an IC_50_ of ~20 µM. In this study, we synthesized a new anti‐inflammatory 4-HAB analogue, F240B, which has an IC_50_ of less than 1 µM. F240B dose-dependently induced autophagy by increasing autophagic flux, LC3 speck formation and acidic vesicular organelle formation. F240B inhibited NACHT, LRR and PYD domain-containing protein 3 (NLRP3) inflammasome activation through autophagy induction. In a mechanistic study, F240B inhibited interleukin (IL)-1β (IL-1β) precursor expression, promoted degradation of NLRP3 and IL-1β, and reduced mitochondrial membrane integrity loss in an autophagy-dependent manner. Additionally, F240B inhibited apoptosis-associated speck-like protein containing a CARD (ASC) oligomerization and speck formation without affecting the interaction between NLRP3 and ASC or NIMA-related kinase 7 (NEK7) and double-stranded RNA-dependent kinase (PKR). Furthermore, F240B exerted *in vivo* anti-inflammatory activity by reducing the intraperitoneal influx of neutrophils and the levels of IL-1β, active caspase-1, IL-6 and monocyte chemoattractant protein-1 (MCP-1) in lavage fluids in a mouse model of uric acid crystal-induced peritonitis. In conclusion, F240B attenuated the NLRP3 inflammasome through autophagy induction and can be developed as an anti-inflammatory agent in the future.

## Introduction

The intracellular sensor, NACHT, LRR, and PYD domain-containing protein 3 (NLRP3) inflammasome, is a multimeric protein complex composed of a sensor NLRP3, an adaptor protein apoptosis-associated speck-like protein (ASC) and an effector caspase-1. Activation of the NLRP3 inflammasome is a two-step process that involves priming and activation. During priming, transcriptional upregulation of NLRP3 and the pro-inflammatory cytokines interleukin (IL)-1β (IL-1β) precursor (proIL-1β) is induced by recognition of pathogen-associated molecular patterns by pattern recognition receptors (1). The major signaling pathways involved in the priming stage of the NLRP3 inflammasome are reactive oxygen species (ROS), the mitogen-activated protein kinase (MAPK) pathway and nuclear factor kappa B (NF-κB)-associated pathways (1, 2). During the activation stage, damage or danger-associated molecular patterns, such as bacterial toxins and extracellular ATP, lead to the assembly of the NLRP3 inflammasome which in turn result in the activation of cysteine protease caspase-1 (1, 3) and the conversion of proIL-1β to mature IL-1β by the active caspase-1.

Since NLRP3 plays a significant role in innate immunity, dysregulated activation results in inflammatory conditions such as atherosclerosis, Type-II diabetes, gout and various neurodegenerative disorders (3). Anti-inflammatory drugs such as colchicines and glucocorticoids, are commonly used to treat NLRP3-related inflammatory diseases, however these drugs exhibit severe side-effects which limits their applications (4). Hence new therapeutics that potentially inhibit NLRP3 with minimal side-effects need to be developed.

Conjugated polyenes occurring in natural products have been shown to exhibit antibacterial, antifungal and antitumor activities (5). Despite their biological significance, the usage of these compounds as therapeutics has been limited because of their availability. Presently, the only known sources of natural polyenes are bacteria and fungi and the amounts of polyenes that can be obtained from them are very low. Chemical synthesis offers an alternative source to these compounds. In our earlier studies, we have successfully synthesized a library of analogues that was derived from a naturally occurring polyene (6–8). We found that one of the analogous, 4-hydroxy auxarconjugatin B (4-HAB), a polyenylpyrrole derivative, inhibits NLRP3 inflammasome activation by inhibiting ASC oligomerization, lysosomal rupture and mitochondrial damage in an autophagy-dependent manner. 4-HAB was also shown to ameliorate uric acid crystal-mediated peritonitis in mice (9).

In the present study, we have synthesized analogs of 4-HAB with the aim of enhancing its anti-NLRP3 inflammasome activity. We found that one of the analogs, 3-butyl-6-[(1E,3E,5E,7E)-8-(3-chloro-1H-pyrrol-2-yl)-1-methyl-1,3,5,7-octatetraen-1-yl]-4-ethoxy-2H-pyran-2-one (F240B), is a more potent NLRP3 inflammasome inhibitor (IC_50_ < 1 µM) than its parent, 4-HAB. Herein we describe the synthesis of F240 and the results for our anti-inflammatory studies.

## Materials and Methods

### General Synthesis Procedures

All chemical reagents and solvents were obtained from Sigma Aldrich, Merck, Alfa Aesar, Fluka or TCI and were used without further purification. Microwave-assisted reactions were performed using the Biotage Initiator microwave synthesizer. TLC were performed on precoated silica plated (Merck silica gel 60, F254) and visualized with UV or stained with phosphomolybdic acid, alkaline potassium permanganate stain. Flash chromatography was performed on silica (Merck, 70-230 mesh). ^1^H, ^13^C and ^31^P NMR spectra were measured on a Bruker AMX 300, 500 or 400 spectrometer (Supporting Information). Chemical shifts were reported in parts per million (δ) relative to the tetramethylsilane standard. Mass spectra were performed on a Finnigan/MAT LCQ mass spectrometer under either electron spray ionization (ESI) or electron impact (EI) techniques.

### Synthesis of 2

Synthesis of 2 was performed according to the procedure reported by Fang et al. (6). The compound was obtained in 28% yield (over 4 steps).

### Synthesis of 3

The crude mixture of 2 was then taken up in DMSO (5 ml), and K_2_CO_3_ (645 mg, 4.68 mmol) and Et_2_SO_4_ (264 µl, 2.06 mmol) were added. The reaction was stirred at room temperature for 2h. The mixture was diluted in ethyl acetate and washed with water 3 times. The organic layer was dried over MgSO_4_, filtered and concentrated. Flash chromatography of the crude mixture (hexane: ethyl acetate = 2:1) gave 3 (257 mg, 65%) as a white solid. ^1^H NMR (500 MHz, CDCl_3_) δ 5.92 (s, 1H), 4.03 (q, *J* = 7.0 Hz, 2H), 2.39 – 2.28 (m, 2H), 2.16 (s, 3H), 1.43 – 1.30 (m, 5H), 1.24 (dq, *J* = 14.4, 7.2 Hz, 2H), 0.83 (t, *J* = 7.3 Hz, 3H). ^13^C NMR (126 MHz, CDCl_3_) δ 165.52, 165.27, 160.49, 105.26, 95.52, 64.51, 29.95, 22.72, 22.34, 20.05, 14.62, 13.79. HRMS (EI) for C_12_H_18_O_3_ calculated 210.1256, found 210.1259.

### Synthesis of 4

To a solution of 3 (743 mg, 3.54 mmol) in dioxane (3 ml) was added SeO_2_ (1.96 g, 17.7 mmol). The reaction was stirred under microwave conditions at 150°C for 30 min. The mixture was then carefully quenched with NaHCO_3_, and the aqueous layer washed three times with CH_2_Cl_2_. Flash chromatography of the crude mixture (hexane:ethyl acetate = 4:1) gave 4 (654 mg, 56%) as a white solid. ^1^H NMR (500 MHz, CDCl_3_) δ 9.48 (s, 1H), 6.91 (s, 1H), 4.24 – 4.06 (m, 2H), 2.53 – 2.36 (m, 2H), 1.48 – 1.33 (m, 5H), 1.27 (dd, *J* = 14.9, 7.4 Hz, 2H), 0.85 (t, *J* = 7.3 Hz, 3H). ^13^C NMR (126 MHz, CDCl_3_) δ 183.34, 162.66, 162.56, 152.31, 115.77, 102.62, 65.46, 29.60, 23.81, 22.49, 14.66, 13.77. HRMS (EI) for C_12_H_16_O_4_ calculated 224.1049, found 224.1049.

### Synthesis of 6

To a solution of 4 (654 mg, 2.92 mmol) in THF (13 ml) was added MeMgBr (3 M in Et_2_O, 1.07 ml, 3.21 mmol). The reaction was stirred at room temperature for 1 h. The mixture was quenched with H_2_O and the aqueous layer washed three times with ethyl acetate. The combined organic layer was dried over MgSO_4_, filtered and concentrated to give crude 5 as a white solid. To a solution of 5 (2.92 mmol) in CH_2_Cl_2_ (20 ml) was added MnO_2_ (3.8 g, 43.8 mmol). The reaction was stirred at room temperature for 12h. The mixture was filtered through celite and concentrated. Flash chromatography of the crude mixture (hexane: ethyl acetate =4:1) gave 6 (608 mg, 88%) as a white solid. ^1^H NMR (500 MHz, CDCl_3_) δ 6.91 (d, *J* = 3.8 Hz, 1H), 4.11 (dt, *J* = 10.9, 5.1 Hz, 2H), 2.51 – 2.38 (m, 5H), 1.41 (dd, *J* = 15.1, 7.8 Hz, 2H), 1.35 (td, *J* = 7.0, 3.9 Hz, 3H), 1.28 (dd, *J* = 14.8, 7.3 Hz, 2H), 0.85 (td, *J* = 7.3, 3.7 Hz, 3H). ^13^C NMR (126 MHz, CDCl_3_) δ 191.47, 163.38, 162.83, 153.39, 114.68, 98.76, 65.33, 29.84, 25.56, 23.77, 22.51, 14.69, 13.72. HRMS (EI) for C_13_H_18_O_4_ calculated 238.1205, found 238.1204.

### Synthesis of 7

To a solution of 6 (608 mg, 2.56 mmol) in CH_2_Cl_2_ (20 ml) at 0°C was added PPh_3_ (1.88 g, 7.16 mmol) and CBr_4_ (1.19 g, 3.58 mmol). The reaction was stirred at room temperature for 2h. The mixture was concentrated and flash chromatography of the crude mixture (hexane: ethyl acetate = 8:1) gave **7** (802 mg, 80%) as a red oil. ^1^H NMR (500 MHz, CDCl_3_) δ 6.35 (s, 1H), 4.11 (q, *J* = 6.9 Hz, 2H), 2.42 (t, *J* = 7.6 Hz, 2H), 2.12 (s, 3H), 1.47–1.37 (m, 5H), 1.35–1.28 (m, 2H), 0.89 (t, *J* = 7.3 Hz, 3H). ^13^C NMR (125 MHz, CDCl_3_) δ 164.3, 164.0, 157.6, 135.4, 108.2, 98.8, 94.2, 64.9, 29.8, 23.2, 22.6, 22.5, 14.7, 13.9. HRMS (EI): for C_14_H_18_O_3_Br_2_ calculated 391.9623; found 391.9640.

### Synthesis of 1

**Scheme 1:**
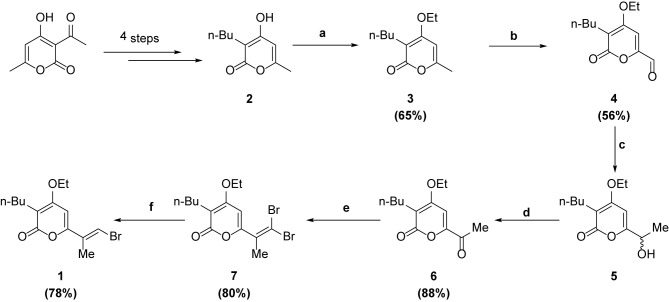
Synthesis of intermediate 1.

To a solution of 7 (802 mg, 2.04 mmol) in CH_2_Cl_2_ (4 ml) was added TEA (1.27 ml, 9.16 mmol) and dimethylphosphite (746 µl, 8.14 mmol). The reaction was stirred at room temperature for 2h. The mixture was diluted with Et_2_O and washed with water 3 times. The organic layer was dried over MgSO_4_, filtered and concentrated. Flash chromatography of the crude mixture (hexane: ethyl acetate = 8:1) gave 1 (502 mg, 78%) as a white solid (**Scheme 1**). ^1^H NMR (400 MHz, CDCl_3_) δ 7.25 (s, 1H), 6.10 (s, 1H), 4.07 (q, *J* = 7.0 Hz, 2H), 2.36 (t, *J* = 7.5 Hz, 2H), 1.99 (s, 3H), 1.42 – 1.33 (m, 5H), 1.31 – 1.20 (m, 2H), 0.83 (t, *J* = 7.2 Hz, 3H). ^13^C NMR (101 MHz, CDCl_3_) δ 164.63, 163.99, 157.04, 131.82, 114.73, 108.47, 94.18, 64.78, 29.99, 23.18, 22.49, 15.69, 14.76, 13.90. HRMS (EI) for C_14_H_19_O_3_^81^Br calculated 316.0497, found 316.0499.

### Synthesis of 8

**Scheme 2:**
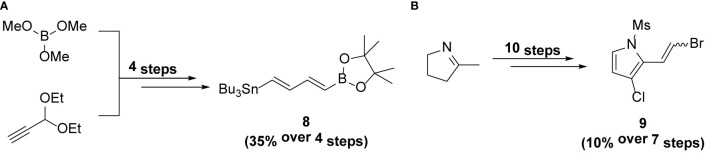
Synthesis of intermediates **(A)** 8 and **(B)** 9.

Compound 8 was prepared from trimethylborate and propargyldehyde diethyl acetal using the procedure reported by Feng et al. (6). It was obtained in 35% yield (over 4 steps) (**Scheme 2**).

### Synthesis of 9

Compound 9 was prepared from 2-methyl-1-pyrroline using the procedure reported by Feng et al. (6). It was obtained in 10% yield (over 7 steps) (**Scheme 2**).

### Synthesis of F240B

**Scheme 3:**
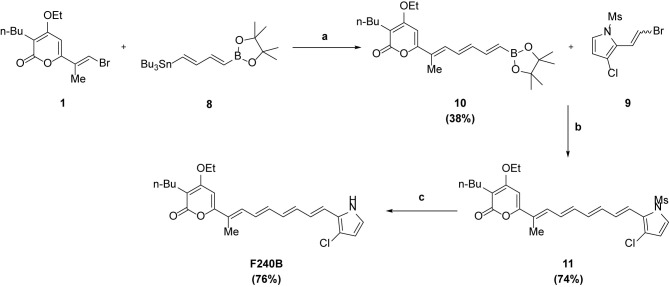
Synthesis of F240B.

A round bottom flask was charged with degassed NMP (1 ml) and 1 (126mg, 0.401 mmol), 8 (283 mg, 0.603 mmol), AsPh_3_ (14.75 mg, 48.2 µmol) and Pd(PPh_3_)_4_ (11 mg, 12 µmol) were added sequentially. The reaction was stirred at room temperature for 6h. The mixture was then diluted in Et_2_O and washed with water 3 times followed by brine. The organic layer was dried over MgSO_4_, filtered and concentrated and then taken up in CH_3_CN. The CH_3_CN layer was then washed with hexane 3 times and then concentrated. Flash chromatography of the crude mixture (hexane:ethyl acetate = 2:1) through a short plug of silica gave 10 (62 mg, 38%) which was used immediately in the subsequent step. To a solution of 9 (64 mg, 0.225 mmol) in THF (0.4 ml) was added AsPh_3_ (4.6 mg, 15 µmol), 10 (62 mg, 0.15 mmol), Pd(PPh_3_)_4_ (2.7 mg, 3 µmol) and 10% KOH (0.17 ml, 0.3 mmol) sequentially. The reaction was stirred at room temperature for 30 min. The mixture was quenched with NH_4_Cl, taken up in ethyl acetate and washed with water 3 times. The organic layer was dried over MgSO_4_, filtered and concentrated. Flash chromatography of the crude mixture (hexane:ethyl acetate = 3:1) over a short plug of silica gave 11 (54 mg, 74%) as a red solid. 11 (164 mg, 0.333 mmol) was then taken up in THF (13 ml) and TBAF (1M in THF, 0.667 ml, 0.667 mmol) was added dropwise at 0℃. The reaction was stirred at room temperature for 1h. The mixture was diluted in ethyl acetate and washed with water 3 times, followed by brine. The organic layer was dried over MgSO_4_, filtered and concentrated. Flash chromatography of the crude mixture (hexane:ethyl acetate = 3:1) over a short plug of silica gel gave F240B (104 mg, 76%) as a red solid (**Scheme 3**). ^1^H NMR (500 MHz, DMSO-*d*_6_) δ 11.5 (br, 1H), 7.04 (d, *J* = 10.7 Hz, 1H), 6.89 (s, 1H), 6.80-6.68 (m, 3H), 6.61 (dd, *J* = 11.3, 15.1 Hz, 1H), 6.53–6.44 (m, 3H), 6.13 (s, 1H), 4.26 (q, *J* = 6.9 Hz, 2H), 2.34 (t, *J* = 7.6 Hz, 2H), 2.03 (s, 3H), 1.41–1.35 (m, 2H), 1.33–1.24 (m, 5H), 0.88 (t, *J* = 7.3 Hz, 3H). ^13^C NMR (125 MHz, DMSO-*d*_6_) δ 165.3, 163.1, 158.9, 138.5, 136.2, 131.6, 131.0, 127.9, 126.0, 125.8, 124.8, 120.4, 120.4, 111.7, 109.1, 105.3, 94.3, 64.7, 29.6, 22.6, 21.8, 14.6, 13.7, 12.3. HRMS (EI): for C_24_H_28_NO_3_Cl calculated 413.1758; found 413.1751.

### Biological Materials

Lipopolysaccharide (LPS) from *Escherichia coli* O111:B4 strain (L2630), N-acetyl-L-cysteine (NAC) (A9165), phorbol 12-myristate 13-acetate (PMA) (79346), acridine orange (AO) (A9231), monodansylcadaverine (MDC) (30432) and 4’,6-diamidino-2-phenylindole (DAPI) (MBD0015) were purchased from Sigma-Aldrich (St. Louis, MO). Macrophage colony-stimulating factor (M-CSF) was purchased from Peprotech (London, UK). ATP (tlrl-atp), nigericin (tlrl-nig), monosodium urate (MSU) (tlrl-msu), nanoparticles of silica dioxide (SiO_2_) (tlrl-sio), pNiFty2-SEAP plasmids (pnifty-seap), pSELECT-GFP-mLC3 plasmids (psetz-gfpmlc3), QUANTI-Blue medium (rep-qb2), poly(dA:dT) (tlrl-patn), muramyldipeptide (MDP) (tlrl-mdp), FLA-ST (flagellin from *Salmonella typhimurium*) (tlrl-stfla) and Pam3CSK4 (tlrl-pms) were purchased from InvivoGen (San Diego, CA). ASC-GFP plasmids were purchased from Genomics (Taipei, Taiean). Antibody against IL-1β (AB-401-NA) was purchased from R&D systems (Minneapolis, MN). Antibodies against caspase-1 (AG-20B-0044) and NLRP3 (AG-20B-0014) were purchased from Adipogen Life Science (San Diego, CA). Antibodies against Actin (sc-47778), ASC (sc-25514-R), cyclooxygenase-2 (COX-2) (sc-376861), phospho-IκB-α (sc-8404) and CRISPR/Cas9 knockout plasmids targeting LC3 (sc-426563 and sc-417828-HDR) were purchased from Santa Cruz Biotechnology (Santa Cruz, CA). Antibodies against phospho-MAPK (#9910) were purchased from Cell Signaling Technology (Beverly, MA). Antibodies against NIMA-related kinase 7 (NEK7) (ab96538) and double-stranded RNA-dependent protein kinase (PKR) (ab184257) were purchased from Abcam (Cambridge, UK). Antibodies against LC3B (NB100-2220) and p62 (#5114) were purchased from Novus Biologicals (Littleton, CO). RPMI-1640 medium (22400089), Mitotracker Deep Red (M22426), Mitotracker Green (M7514), MitoSOX (M36008), disuccinimidyl suberate (21655), CM-H_2_DCFDA (C6827), ELISA kits of IL-1β (88-7013-88), IL-6 (88-7064-88), tumor necrosis factor alpha (TNF-α) (88-7324-88) monocyte chemoattractant protein-1 (MCP-1) (88-7503-88), and antibodies against Gr1 (12-5931-83) and CD45 (11-0451-82) were purchased from Thermo Fisher Scientific (Waltham, MA). The CytoScan LDH Cytotoxicity Assay kit was purchased from G-Bioscience (St. Louis, MO). MCC950 was purchased from TargetMol (Wellesley Hills, MA).

### Cell Culture

J774A.1 macrophages, RAW264.7 macrophages and THP-1 monocytes were purchased from the American Type Culture Collection (Rockville, MD). LC3-knockout J774A.1 macrophages were established by CRISPR/Cas9 knockout plasmids in our previous studies (9, 10). Briefly, J774A.1 macrophages were transfected with CRISPR/Cas9 knockout plasmids targeting LC3 and the cells were selected by puromycin-containing medium. The protein expression levels of LC3 were checked by Western blot. J774A.1 NF-κB reporter cells were established by the pNiFty2-SEAP plasmids in our previous study (10). LC3-GFP and ASC-GFP expressing J774A.1 macrophages were established by transient transfection of pSELECT-GFP-mLC3 plasmids and ASC-GFP plasmids, respectively. To generate bone marrow-derived macrophages (BMDM), marrow was collected from femur and tibia of C57BL/6 mice and cultured for 7 days in culture medium containing 30 ng/ml M-CSF. To generate THP-1 macrophages, THP-1 monocytes were cultured for 2 days in culture medium containing 50 nM PMA. Human primary peripheral blood mononuclear cells (PBMCs) were prepared from whole blood from healthy volunteers. Briefly, free collected whole blood was separated by density gradient centrifugation using Histopaque-1077. Experimental protocols for whole blood collection were performed in accordance with the guidelines and regulations provided and accepted by the Institutional Review Board of the Tri-Service General Hospital, National Defense Medical Center. All cells were cultured in RPMI-1640 medium with 10% heat-inactivated foetal bovine serum at 37°C in a 5% CO_2_ incubator.

### Bacterial Strains and Infection

*Salmonella* (ATCC 14028) were purchased from the American Type Culture Collection (Rockville, MD), and cultured on *Salmonella Shigella* agar (Creative, TW) at 37°C in a 5% CO_2_ incubator. The number of viable *Salmonella* was determined by a colorimeter (M6+ Colorimeter, Metertech). J774A.1 macrophages were primed with 1 µg/ml LPS for 5 h followed by treated with 1 µM F240B for 3 h. Cells were infected 2 h with *Salmonella* at a multiplicity of infection (MOI) of 20.

### Lactate Dehydrogenase Release Assay

To determine the cytotoxicity of F240B, J774A.1 macrophages were incubated with 0.1-1 µM F240B, lysis buffer (maximum LDH release) or 10% H_2_O (spontaneous LDH release) for 3.5 or 24.5 h. Cytotoxicity was determined by measuring the LDH levels in culture medium using the CytoScan LDH Cytotoxicity Assay kit as described previously (9). The cytotoxicity % was calculated as 100 X (sample OD − spontaneous OD)/(maximum OD - spontaneous OD).

### Autophagy Analysis

J774A.1 macrophages were incubated for 3-24 h with 1 µM F240B, for 24 h with 0.1-1 µM F240B or for 4 h with 0.1 µM rapamycin. The protein expression levels of LC3 and p62 were measure by Western blotting. For LC3 speak formation assay, GFP-LC3 expressed J774A.1 macrophages were incubated for 3 h with 1 µM F240B or for 4 h with 0.1 µM rapamycin. The cells were stained by 0.5 µg/ml DAPI for 10 min in the dark and fixed in 4% paraformaldehyde for 0.5 h. The LC3-GFP speak formation was measured by confocal microscopy. For AO and MDC staining, J774A.1 macrophages were incubated for 3 h with 1 µM F240B or for 4 h with 0.1 µM rapamycin. The cells were stained with 1 µg/ml AO or 50 nM MDC at 37°C for 10 min and fixed in 4% paraformaldehyde for 0.5 h. The fluorescent signals were acquired by confocal microscopy.

### Activation of the NLRP3 Inflammasome and Inflammatory Mediator

For the NLRP3 inflammasome activation, cells were incubated with 1 µg/ml LPS for 5 h followed by incubated with 0.1–1 µM F240B for 0.5 h or 3 h. Cells then incubated with 5 mM ATP for 0.5 h, 10 μM nigericin for 0.5 h, 100 μg/ml MSU for 24 h or 100 μg/ml SiO_2_ for 24 h. The levels of IL-1β or TNF-α in the supernatants were measured by ELISA. The levels of IL-1β, caspase-1, NLRP3 and ASC in the supernatants were measured by Western blotting as described previously (9). For inflammatory mediator expression, cells were incubated with 0.1–1 µM F240B for 0.5 h followed by incubated with 1 µg/ml LPS for 24 h. The COX-2 expression was analysed by Western blotting, TNF-α and IL-6 expressions were analysed by ELISA and NO production was analysed by Griess reaction.

### Mitochondrial Damage Assay

J774A.1 macrophages or LC3-knockout J774A.1 macrophages were incubated with or without 1 μg/ml LPS for 5 h followed by incubation with or without 1 µM F240B for 0.5 h in the presence or absence of 5 mM 3-MA. Cells were then incubated with 5 mM ATP for 0.5 h. To analyze mitochondrial integrity, cells were stained with 25 nM MitoTracker Deep Red (for intact mitochondria) and 25 nM MitoTracker Green (for total mitochondria) for 15 min. To analyze mitochondrial ROS, cells were stained with 5 μM MitoSOX (specific mitochondrial ROS indicator) for 15 min. The fluorescent signals were acquired by confocal microscope.

### ASC Oligomerization and Speck Formation

To detect ASC oligomerization, J774A.1 macrophages were incubated with 1 µg/ml LPS for 5 h followed by incubation with with 1 µM F240B for 3 h. Cells were then incubated with 5 mM ATP for 0.5 h and lysed by lysis buffer. After centrifuge, the pellets were cross-linked with 2 mM disuccinimidylsuberate at 37°C for 30 min and subsequent analyzed by Western blotting (9). To detect ASC speck formation, ASC-expressing J774A.1 macrophages were incubated with 1 µg/ml LPS for 5 h followed by incubated with 1 µM F240B for 3 h. Cells were then incubated with 5 mM ATP for 0.5 h. Cells were fixed in 4% paraformaldehyde for 0.5 h and the fluorescent signals were acquired by confocal microscope.

### Immunoprecipitation-Western Blotting Assay

To detect the binding between NLRP3 and ASC, NEK7 and PKR, J774A.1 macrophages were incubated with 1 µg/ml LPS for 5 h followed by incubation with 1 µM F240B or 0.1 µM MCC950 for 3 h. Cells were then incubated with 5 mM ATP for 0.5 h and lysed by lysis buffer. Prepared a 500 μl mixture containing 500 μg protein and 1 μg NLRP3 or ASC antibody. Incubated the mixture with gentle rocking at 4°C for overnight followed by adding 20 μl protein A/G agarose bead and gentle rocking at 4°C for additional 1 h. After centrifuge, the supernatant was removed and the pellet was subsequent analyzed by Western blotting against NLRP3, NEK7, and PKR, respectively.

### Mice Model

C57BL/6JNal mice (male, 8-week-old) were purchased from The National Laboratory Animal Center (Taipei, Taiwan). The mice were housed in the animal center of National Ilan University under standard and controlled environment. The studies were performed with the approval and regulation of Institutional Animal Care and Use Committee of the National Ilan University (approval number: No. 102-40). The mice were randomly divided into four groups: Control group: intraperitoneal injection (i.p.) of 0.5% DMSO in sterile PBS (200 μl) at 0, 24 and 48 h; i.p. injection of sterile PBS (0.5 ml) at 1 and 49 h, n=6. MSU group: i.p. injection of 0.5% DMSO in sterile PBS (200 μl) at 0, 24 and 48 h; i.p. injection of sterile MSU crystals (3 mg in 0.5 ml PBS) at 1 and 49 h, n=8. F240B +MSU group: i.p. injection of F240B (20 mg/kg body weight) at 0, 24 and 48 h; i.p. injection of sterile MSU crystals (3 mg in 0.5 ml PBS) at 1 and 49 h, n=6. Colchicine+MSU group: i.p. injection of colchicine (1 mg/kg body weight) at 48 h; i.p. injection of sterile MSU crystals (3 mg in 0.5 ml PBS) at 1 and 49 h, n=6. Mice were euthanized at 53 h and the peritonea were lavaged with 3 ml ice-cold PBS. The absolute number of cells was counted and neutrophil peritoneal influx were quantified by Gr-1 and CD45 staining and analysed by flow cytometry. The expression levels of cytokines in peritoneal lavage fluids were measured by ELISA.

### Statistical Analysis

Because of the sample size is small and the data is not normal distribution in this study, the non-parametric Mann-Whitney U-test was used to analyze changes between two groups. All statistical analyses were performed using Prism v5.0 (Graphpad software). *P* values less than 0.05 were considered to be statistically significant.

## Results

### F240B Synthesis

(3-butyl-6-[(1E,3E,5E,7E)-8-(3-chloro-1H-pyrrol-2-yl)-1-methyl-1,3,5,7-octatetraen-1-yl]-4-ethoxy-2H-pyran-2-one) was synthesized according to the procedure reported previously (6, 11).

Reagents and conditions: (a) Et_2_SO_4_, K_2_CO_3_, DMSO, r.t., 2h; (b) SeO_2_, dioxane, m.w. 160°C, 30 min; (c) MeMgBr, THF, r.t., 1h; (d) MnO_2_, DCM, r.t., 12h; (e) CBr_4_, PPh_3_, DCM, 0°C, 2h; (f) (CH_3_O)_2_PHO, TEA, DCM, r.t., 2h.

Intermediates 8 and 9 were synthesized using the procedure reported previously (6, 11).

Reagents and conditions: (a) Pd_2_dba_3_, AsPh_3_, NMP, r.t., 6h; (b) Pd_2_dba_3_, AsPh_3_, KOH, THF, r.t., 1h; (c) TBAF, THF, r.t., 1h.

F240B was synthesized *via* Stille coupling of 2 and 3 using Pd_2_dba_3_, followed by a Suzuki coupling with 4 using Pd_2_dba_3_ and KOH to initially form 11 and 12 in 38% and 74% yield respectively. Demesylation using TBAF in THF gave F240B in 76% yield. The ^1^H NMR and ^13^C NMR spectra of 3 (online resource **Figures 1** and **2**), 4 (online resource **Figures 3** and **4**), 6 (online resource **Figures 5** and **6**), 7 (online resource **Figures 7** and **8**), 1 (online resource **Figures 9** and **10**) and F240B (online resource **Figures 11** and **12**) have been provided in the online resource.

**Figure 1:**
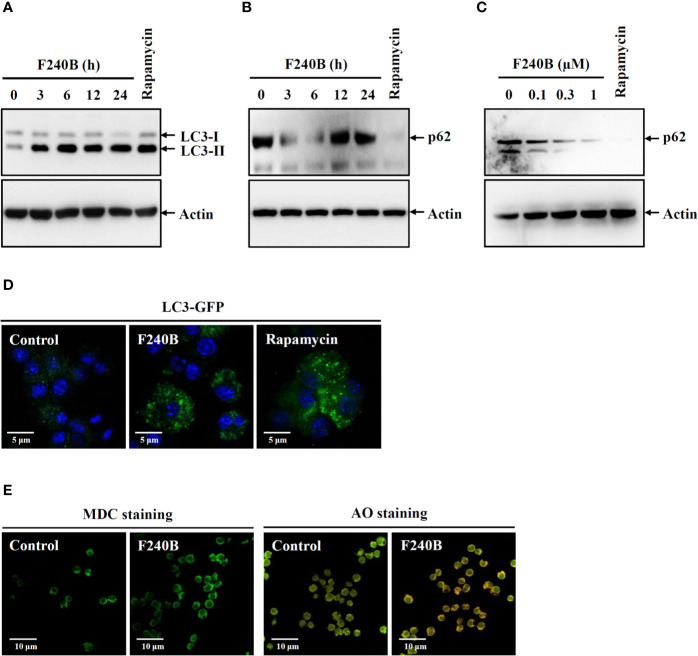
F240B induces autophagy. **(A, B)** J774A.1 macrophages were incubated for 3–24 h with 0.3 µM F240B or for 4 h with 0.1 µM rapamycin. The levels of LC3 **(A)** and p62 **(B)** in the lysates were measured by Western blotting. **(C)** J774A.1 macrophages were incubated for 3 h with 0.1–1 µM F240B or for 4 h with 0.1 µM rapamycin. The levels of p62 in the lysates were measured by Western blotting. **(D)** GFP-LC3 expressed J774A.1 macrophages were incubated for 3 h with 1 µM F240B or for 4 h with 0.1 µM rapamycin. The LC3-GFP speck formation was measured by confocal microscopy. **(E)** J774A.1 macrophages were incubated for 3 h with 1 µM F240B. The cells were stained with 50 nM MDC or 1 µg/ml AO and the fluorescent signals were acquired by confocal microscopy.

**Figure 2:**
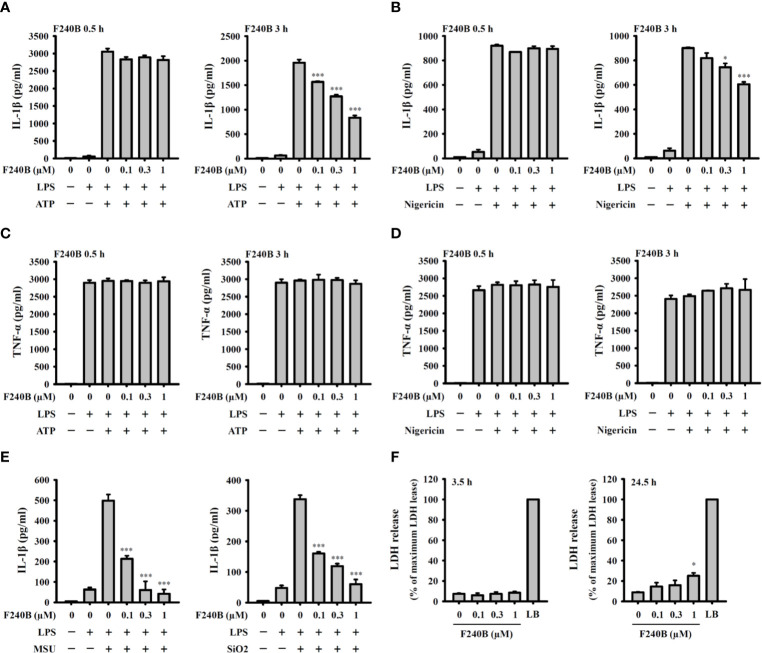
F240B inhibits the NLRP3 inflammasome-derived IL-1β secretion in J774A.1 macrophages. J774A.1 macrophages were incubated with 1 µg/ml LPS for 5 h followed by incubated with F240B for 0.5 h or 3 h. Cells then incubated with 5 mM ATP for 0.5 h **(A, C)**, 10 μM nigericin for 0.5 h **(B, D)**, 100 μg/ml MSU or 100 μg/ml SiO_2_ for 24 h **(E)**. The levels of IL-1β and TNF-α in the supernatants were measured by ELISA. (F) J774A.1 macrophages were incubated with F240B for 3.5 h or 24 h, and cytotoxicity was analyzed by LDH release. The data are expressed as the mean ± SD of three separate experiments. * and *** indicate a significant difference at the level of *p* < 0.05 and *p* < 0.001, respectively, compared to LPS+ATP **(A, C)**, LPS+Nigericin **(B, D)**, LPS+MSU or LPS+SiO_2_ **(E)** or control **(F)**.

**Figure 3:**
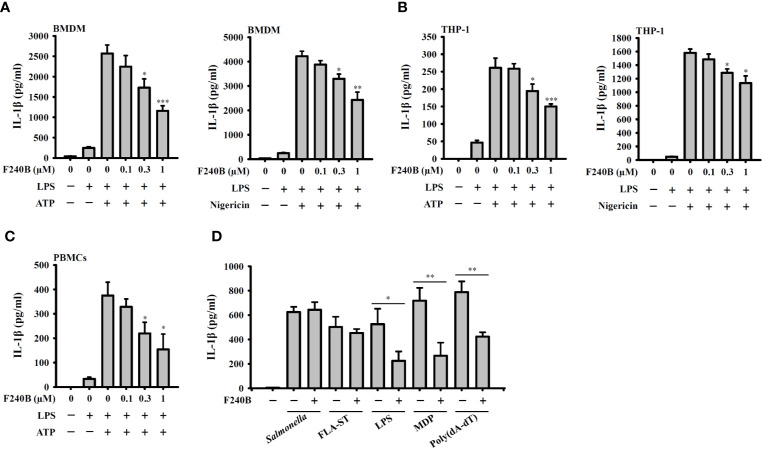
Effect of F240B on inflammasome activation. **(A)** BMDM, **(B)** THP-1 macrophage or **(C)** PBMCs were incubated with 1 µg/ml LPS for 5 h followed by incubated with F240B for 3 h. Cells then incubated with 5 mM ATP or 10 μM nigericin for 0.5 h. The levels of IL-1β in the supernatants were measured by ELISA. **(D)** LPS-primed or Pam3CSK4-primed (for LPS transfection only) J774A.1 macrophages were incubated for 3 h with 1 μM F240B followed by transfection with poly(dA/dT) (2 μg/ml), FLA-ST (1 μg/ml), MDP (10 μg/ml) or LPS (2 μg/ml) for 6 h, or by *Salmonella* infection (20 MOI) for 2 h. The levels of IL-1β in the supernatants were measured by ELISA. The data are expressed as the mean ± SD of three separate experiments. *, ** and *** indicate a significant difference at the level of *p* < 0.05, *p* < 0.01 and *p* < 0.001, respectively, compared to LPS+ATP or LPS+Nigericin **(A–C)** or as indicated **(D)**.

**Figure 4:**
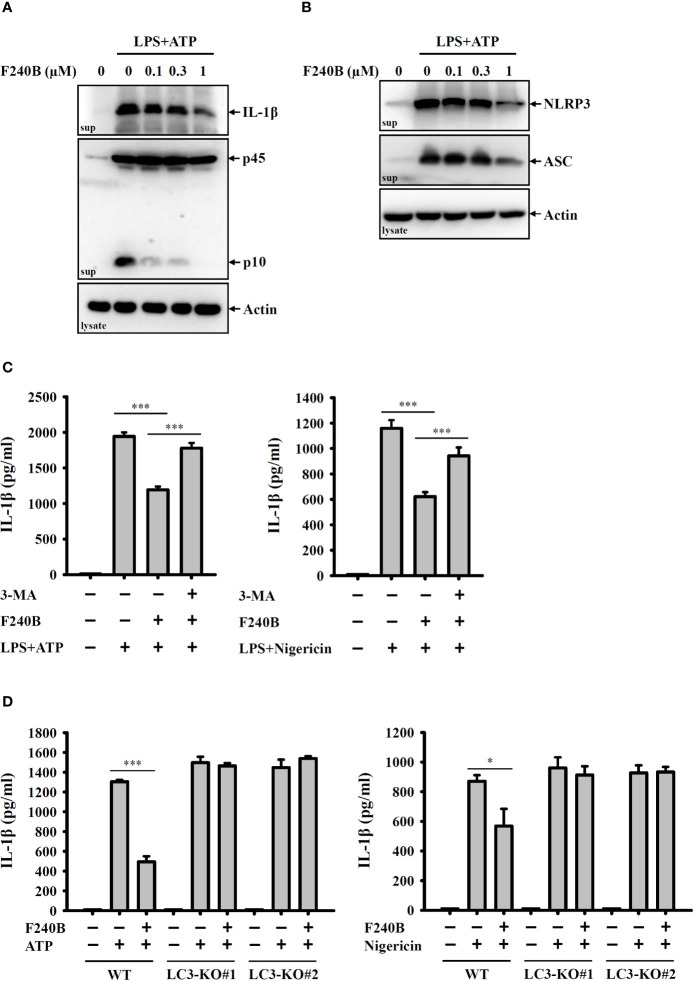
F240B inhibits the NLRP3 inflammasome through autophagy induction. **(A, B)** J774A.1 macrophages were incubated with 1 µg/ml LPS for 5 h followed by incubated with F240B for 3 h. Cells then incubated with 5 mM ATP for 0.5 h. The levels of IL-1β and caspase-1 **(A)** or NLRP3 and ASC **(B)** in the supernatants were measured by Western blotting. **(C)** J774A.1 macrophages were incubated with 1 µg/ml LPS for 5 h followed by incubated with 1 µM F240B in the presence or absence of 5 mM 3-MA for 3 h. Cells then were incubated with 5 mM ATP or 10 μM nigericin for 0.5 h. The levels of IL-1β in the supernatants were measured by ELISA. **(D)** Wild-type and LC3-knockout J774A.1 macrophages were incubated with 1 µg/ml LPS for 5 h followed by incubated with 1 µM F240B for 3 h. Cells then incubated with 5 mM ATP or 10 μM nigericin for 0.5 h. The levels of IL-1β in the supernatants were measured by ELISA. The data are expressed as the mean ± SD of three separate experiments. * and *** indicate a significant difference at the level of *p* < 0.05 and *p* < 0.001, respectively.

**Figure 5:**
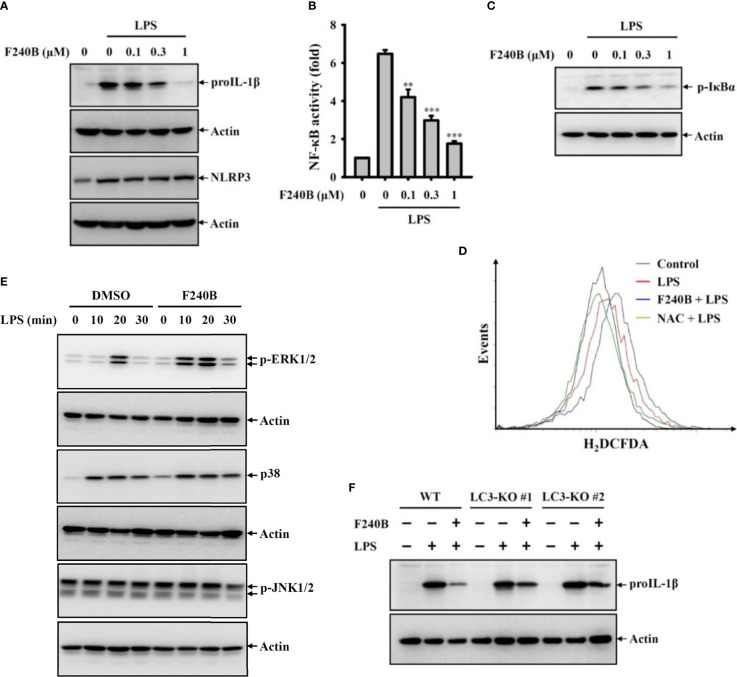
Activation of autophagy by F240B inhibits NF-κB activation and proIL-1β expression. **(A)** J774A.1 macrophages were incubated with F240B for 0.5 h followed by incubated with 1 µg/ml LPS for 6 h. The levels of proIL-1β and NLRP3 in the cell lysates were measured by Western blotting. **(B)** J-Blue cells were incubated with F240B for 0.5 h followed by incubated with 1 µg/ml LPS for 24 h. The NF-κB transcriptional activity was measured by NF-κB reporter assay. **(C, D)** J774A.1 macrophages were incubated with F240B (1 µM for ROS assay) for 0.5 h followed by incubated with 1 µg/ml LPS for 10 min. The phosphorylation levels of IκBα in the cell lysates were measured by Western blotting **(C)**, and the intracellular ROS production was analysed by H_2_DCFDA staining **(D)**. **(E)** J774A.1 macrophages were incubated with 1 µM F240B for 0.5 h followed by incubated with 1 µg/ml LPS for 10-30 min. The phosphorylation levels of ERK1/2, JNK1/2 and p38 in the cell lysates were measured by Western blotting. **(F)** Will-type or LC3-knockout J774A.1 macrophages were incubated with1 µM F240B for 0.5 h followed by incubated with 1 µg/ml LPS for 6 h. The levels of proIL-1β in the cell lysates were measured by Western blotting. ** and *** indicate a significant difference at the level of *p* < 0.01 and *p* < 0.001, respectively compared to LPS.

**Figure 6:**
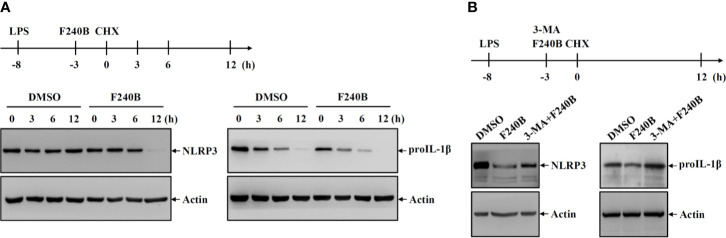
Activation of autophagy by F240B reduced the protein stability of NLRP3 and proIL-1β. **(A)** J774A.1 macrophages were incubated with 1 µg/ml LPS for 5 h followed by incubated with 1 µM F240B or 0.1% DMSO for 3 h. The cells were then incubated with 30 µg/ml CHX for 3-12 h. The levels of NLRP3 and proIL-1β in the cell lysates were measured by Western blotting. **(B)** J774A.1 macrophages were incubated with 1 µg/ml LPS for 5 h followed by incubated with 1 µM F240B for 3 h in the presence or absence of 5 mM 3-MA. The cells were then incubated with 30 µg/ml CHX for 12 h. The levels of NLRP3 and proIL-1β in the cell lysates were measured by Western blotting.

**Figure 7:**
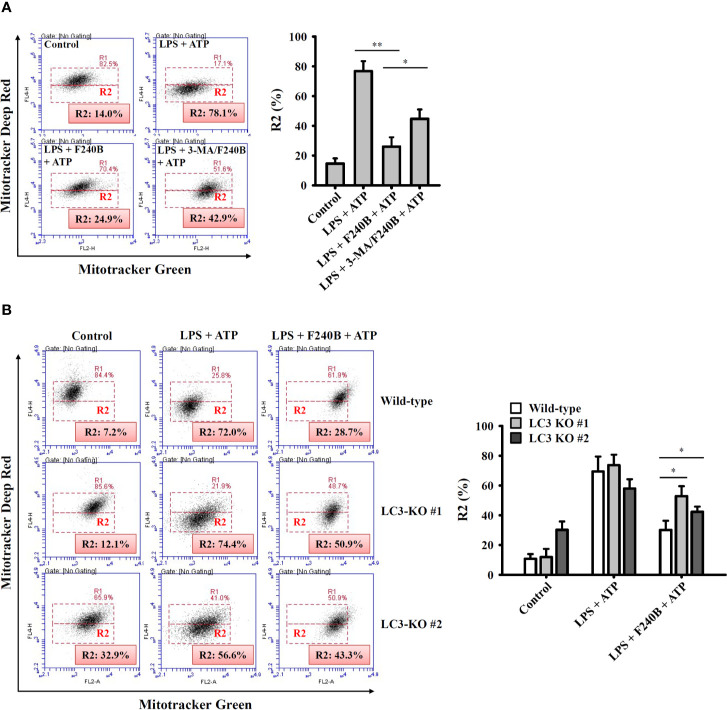
Activation of autophagy by F240B limits mitochondrial integrity loss. **(A)** J774A.1 macrophages were incubated with 1 µg/ml LPS for 5 h followed by incubated with 1 µM F240B in the presence or absence of 5 mM 3-MA for 3 h. Cells then incubated with 5 mM ATP for 0.5 h. **(B)** Wild-type or LC3-knockout J774A.1 macrophages were incubated with 1 µg/ml LPS for 5 h followed by incubated with 1 µM F240B for 3 h. Cells then incubated with 5 mM ATP for 0.5 h. The mitochondrial membrane integrity was measured by staining with MitoTracker Deep Red and MitoTracker Green. The percentage of cells with mitochondrial membrane integrity loss are expressed as the mean ± SD of three separate experiments. * and ** indicate a significant difference at the level of *p* < 0.05 and *p* < 0.01, respectively.

**Figure 8:**
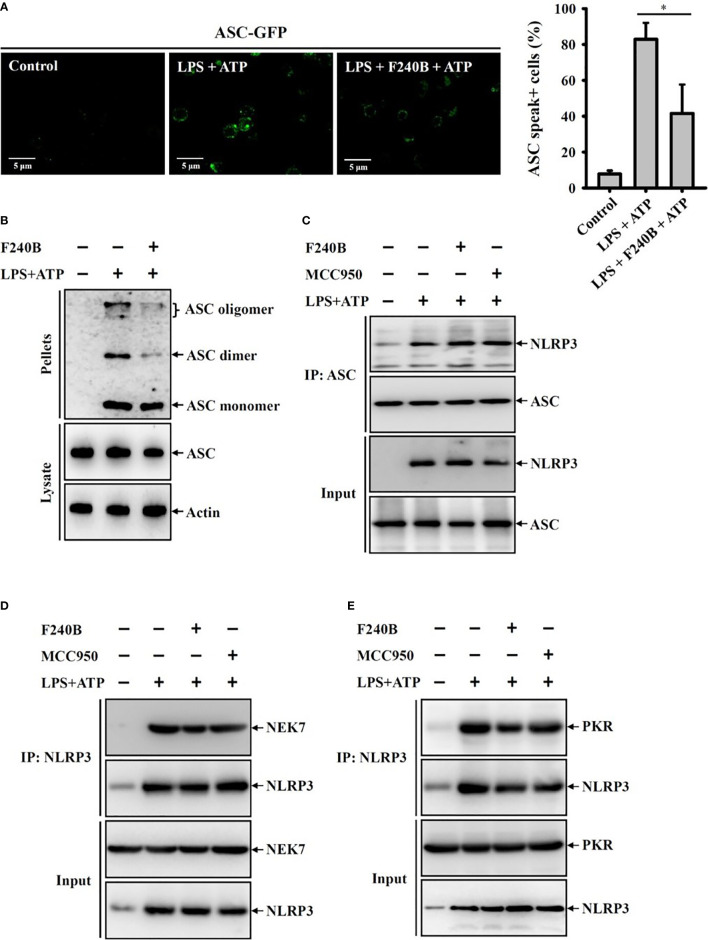
F240B inhibits ASC oligomerization. **(A)** ASC-GFP expressed J774A.1 macrophages or **(B)** J774A.1 macrophages were incubated with 1 µg/ml LPS for 5 h followed by incubated with 1 µM F240B for 3 h. Cells then incubated with 5 mM ATP for 0.5 h. The ASC speck formation was analyzed by fluorescent microscope **(A)**, or the cell lysates were crosslinked by disuccinimidyl suberate and ASC oligomerization was analyzed by Western blotting **(B)**. **(C–E)** J774A.1 macrophages were incubated with 1 µg/ml LPS for 5 h followed by incubated with 1 µM F240B or 0.1 µM MCC950 for 3 h. Cells then incubated with 5 mM ATP for 0.5 h. The interaction between NLRP3 with ASC **(C)**, NEK7 **(D)** or PKR **(E)** were analyzed by immunoprecipitation and Western blotting assay. The percentage of ASC speck positive cells are expressed as the mean ± SD of three separate experiments. * indicates a significant difference at the level of *p* < 0.05.

**Figure 9:**
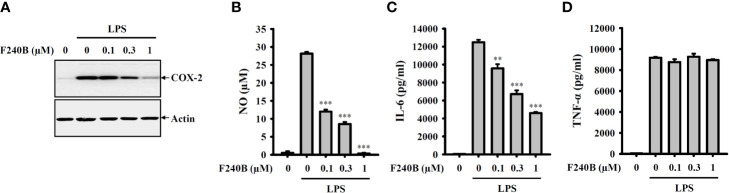
F240B inhibits NO, COX-2 and IL-6 expression. RAW264.7 macrophages were incubated with F240B for 0.5 h followed by incubated with 1 µg/ml LPS for 24 h. The levels of COX-2 **(A)** in the cell lysates were measured by Western blotting, and the levels of NO **(B)**, IL-6 **(C)** and TNF-α **(D)** in the supernatants were measured by ELISA. The data are expressed as the mean ± SD of three separate experiments. ** and *** indicate a significant difference at the level of *p* < 0.01 and *p* < 0.001, respectively, compared to LPS.

**Figure 10:**
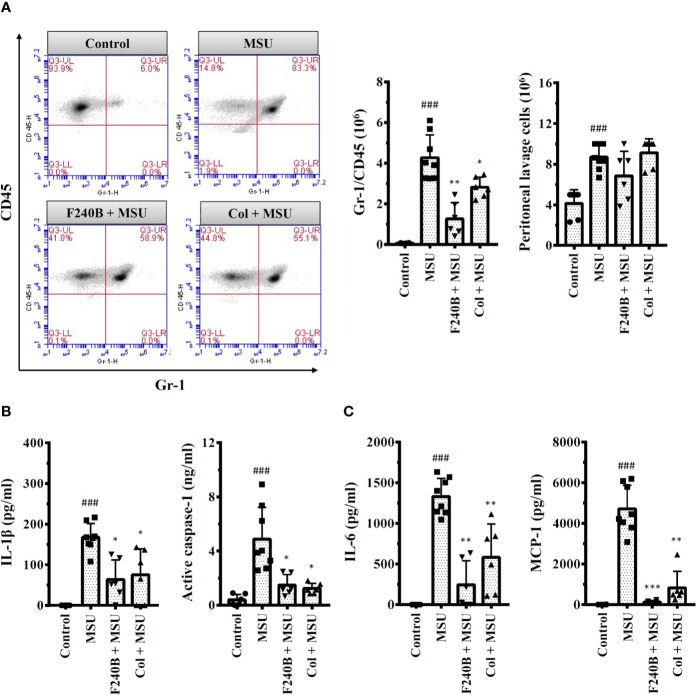
F240B inhibits the NLRP3 inflammasome and inflammation in a mouse model of gouty inflammation. **(A)** Neutrophil and peritoneal lavage cells influx were quantified by Gr-1 and CD45 staining and cell count, respectively. **(B, C)** The expression levels of IL-1β, active caspase-1, IL-6 and MCP-1 in the peritoneal lavage fluids were measured by ELISA. The ELISA data expressed as mean ± SD of three separate experiments. *, ** and *** indicate a significant difference at the level of *p* < 0.05, *p* < 0.01 and *p* < 0.001, respectively, compared to MSU crystal-injected mice. ### indicates a significant difference at the level of *p* < 0.001 compared to control mice.

**Figure 11:**
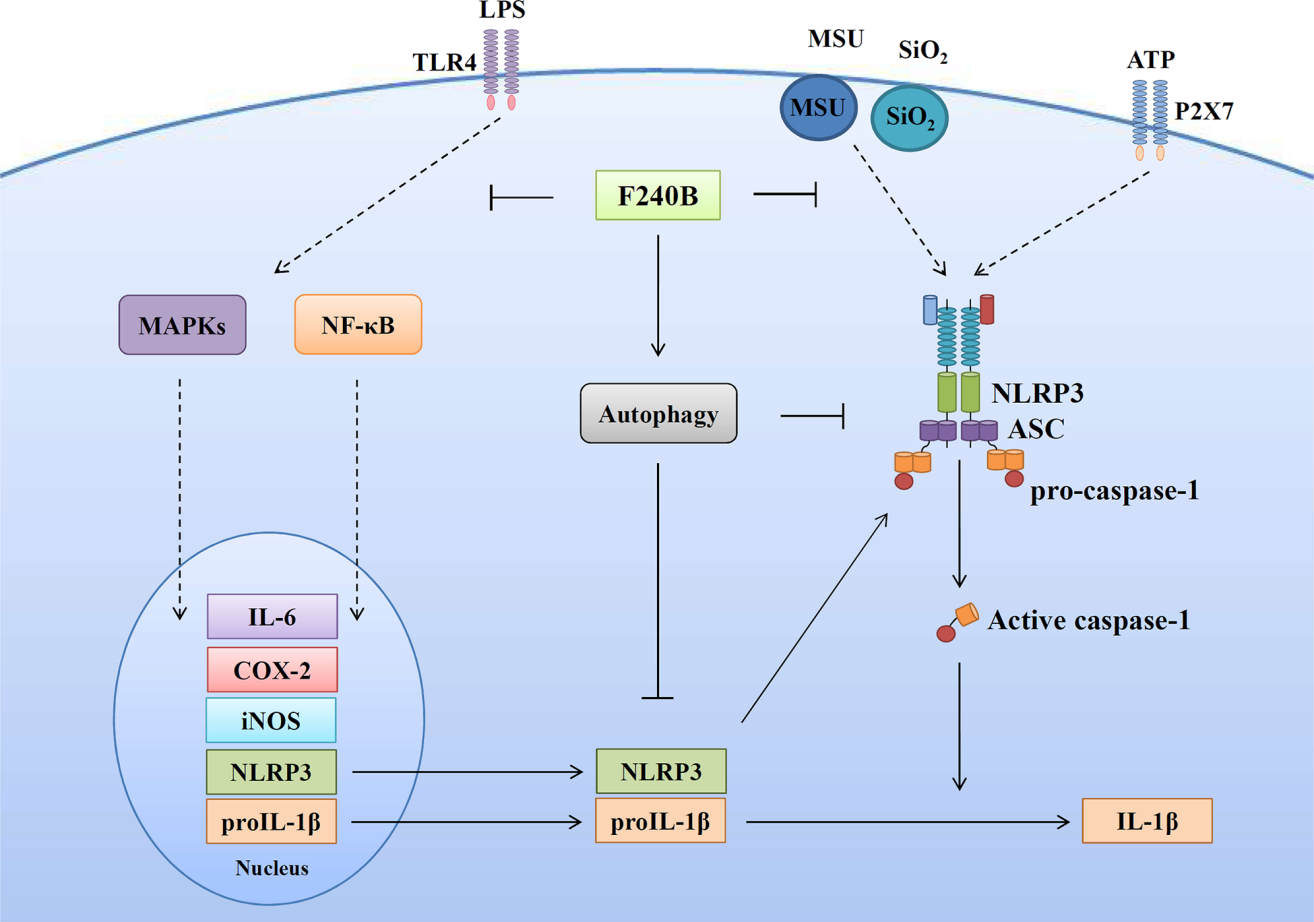
Overview of the putative mechanisms by which F240B inhibited the NLRP3 inflammasome.

### F240B Induces Autophagy

Autophagy is the cellular protective mechanism that inhibits the NLRP3 inflammasome (12). To investigate the effect of F240B on autophagy induction, J774A.1 macrophages were incubated for 3-24 h with 0.3 µM F240B or for 4 h with 0.1 µM rapamycin (an autophagy inducer). The expression levels of the autophagy markers LC3 and p62 in the lysates were measured by Western blotting. We found that the LC3-II expression level was increased by rapamycin or F240B after 3 h of treatment and extended to 24 h (**Figure 1A**). The p62 protein expression level was reduced by rapamycin or F240B after 3 h and 6 h of treatment and recovered after 12 h of treatment, indicating autophagic flux (**Figure 1B**). In addition, incubation of the cells with 0.1–1 µM F240B for 3 h reduced p62 protein expression in a dose-dependent manner (**Figure 1C**). Furthermore, 1 µM F240B treatment for 3 h or 0.1 µM rapamycin treatment for 4 h induced autophagosome (LC3-GFP specks) formation in J774A.1 macrophages that stably expressed GFP-LC3 fusion protein (**Figure 1D**). To confirm the induction of autophagy by F240B, J774A.1 macrophages were incubated for 3 h with 1 µM F240B. The cells were stained with the fluorescent autophagy indicators MDC and AO, and the fluorescent signals were acquired by confocal microscopy. We found that the fluorescent signals of MDC and AO were significantly increased by F240B treatment (**Figure 1E**). These results indicate that F240B is a potent autophagy inducer in macrophages.

### F240B Inhibits the NLRP3 Inflammasome-Derived IL-1β Secretion

Since F240B treatment for 3 h significantly induced autophagy in macrophages (**Figure 1**), we asked whether F240B could inhibit the NLRP3 inflammasome through autophagy induction. To address this hypothesis, LPS-primed J774A.1 macrophages were incubated for 0.5 h (no autophagy induction) or 3 h (autophagy induced) with F240B, followed by treatment with the NLRP3 activator ATP or nigericin for 0.5 h. NLRP3 inflammasome activation was analysed by measuring IL-1β secretion. We found that LPS priming did not significantly induce IL-1β secretion, and ATP (**Figure 2A**) or nigericin (**Figure 2B**) significantly induced IL-1β secretion. Notably, we demonstrated that 3 h but not 0.5 h F240B treatment reduced IL-1β secretion in a dose-dependent manner in ATP- (**Figure 2A**) and nigericin-activated cells (**Figure 2B**). Under the same conditions, neither 0.5 h nor 3 h F240B treatment affected TNF-α secretion, which was independent of the NLRP3 inflammasome (**Figures 2C, D**). In addition, F240B also reduced IL-1β secretion mediated by MSU and SiO_2_, which are also NLRP3 inflammasome activators (**Figure 2E**). To rule out the possibility that F240B-mediated IL-1β inhibition was due to cytotoxic effects, we further investigated the cytotoxicity of F240B in J774A.1 macrophages by LDH release assay. F240B treatment for 3.5 h did not increase LDH release, but F240B slightly increased LDH release at 1 µM after 24.5 h of treatment (**Figure 2F**). These results indicated that F240B-inhibited IL-1β secretion was not due to a reduction in cell viability. The inhibitory activity of F240B on IL-1β was confirmed in BMDMs, human THP-1 macrophages and PBMCs as treatment reduced the IL-1β levels in ATP- or nigericin-activated BMDMs (**Figure 3A**), THP-1 macrophages (**Figure 3B**) and PBMCs (**Figure 3C**). In addition, we investigate whether F240B could inhibits the activation of other inflammasomes. We found that F240B reduced IL-1β secretion in J774A.1 macrophages transfected with LPS, MDP or poly(dA:dT), which activates non-canonical-, NLRP3- and the absent in melanoma 2 (AIM2)-inflammasome, respectively (**Figure 3D**). However, F240B did not reduce the IL-1β secretion in J774A.1 macrophages infected with *Salmonella* or transfected with FLA-ST, which activate NLR family CARD domain containing 4 (NLRC4) inflammasome (**Figure 3D**). These results indicated that F240B not only inhibited the NLRP3 inflammasome but also inhibited non-canonical- and AIM2-inflammasome.

### F240B Inhibits the NLRP3 Inflammasome Through Autophagy Induction

To confirm the inhibitory activity of F240B on the NLRP3 inflammasome, we investigated the effect of F240B on IL-1β secretion and caspase-1 activation using Western blotting. We found that F240B treatment for 3 h significantly reduced ATP-induced IL-1β secretion and caspase-1 activation in LPS-primed J774A.1 macrophages (**Figure 4A**). Activation of the NLRP3 inflammasome induces caspase-1-dependent cell death (pyroptosis) that is characterized by the loss of cell membrane integrity. ATP treatment significantly induced the extracellular release of NLRP3 and ASC in LPS-primed J774A.1 cells, indicating the loss of cell membrane integrity (**Figure 4B**). F240B treatment for 3 h significantly reduced the extracellular release of ASC and NLRP3 (**Figure 4B**), indicating that F240B reduced pyroptosis. To investigate the involvement of autophagy in F240B-mediated NLRP3 inflammasome inhibition, the autophagy inhibitor 3-MA was used to test whether autophagy inhibition abolished F240B-mediated IL-1β downregulation. We found that 3-MA preincubation abolished F240B-mediated IL-1β downregulation in ATP- or nigericin-activated cells (**Figure 4C**). The role of autophagy in F240B-mediated NLRP3 inflammasome inhibition was confirmed by LC3-knockout in J774A.1 macrophages, as F240B did not inhibit IL-1β secretion in ATP- or nigericin-activated LC3-knockout cells (**Figure 4D**). These results indicate that F240B inhibits the NLRP3 inflammasome through autophagy induction.

### Activation of Autophagy by F240B Inhibits NF-κB Activation and ProIL-1β Expression

Induction of proIL-1β and NLRP3 proteins by LPS-mediated priming signaling is the crucial step for NLRP3 inflammasome activation (2). We found that LPS treatment significantly induced proIL-1β and NLRP3 expression in J774A.1 macrophages; notably, F240B significantly reduced proIL-1β but not NLRP3 expression (**Figure 5A**). To dissect the underlying molecular mechanisms of F240B-mediated proIL-1β inhibition, the important priming signals induced by LPS were evaluated. We found that LPS treatment significantly increased NF-κB transcriptional activity as analyzed by the NF-κB reporter assay, and this effect was inhibited by F240B in a dose-dependent manner (**Figure 5B**). F240B also inhibited the phosphorylation level of IκBα in LPS-activated J774A.1 macrophages, confirming that F240B inhibits NF-κB (**Figure 5C**). In addition, we investigated the effect of F240B on LPS-mediated ROS production, which is an upstream priming signal that regulates proIL-1β expression in J774A.1 macrophages (2). We found that LPS treatment significantly increased intracellular ROS production, as analyzed by staining with the ROS indicator H_2_DCFDA; however, F240B did not reduce ROS production in J774A.1 macrophages (**Figure 5D**). Moreover, F240B also did not affect the phosphorylation levels of ERK1/2, JNK1/2 or p38 (**Figure 5E**), which are also priming signal in LPS-activated J774A.1 macrophages (2). Finally, we found that F240B-mediated proIL-1β downregulation was associated with autophagy induction, as the inhibitory effect of F240B on proIL-1β was reduced in LC3 knockout J774A.1 macrophages (**Figure 5F**).

### Activation of Autophagy by F240B Reduced the Protein Stability of NLRP3 and ProIL-1β

It has been demonstrated that autophagy is involved in the cellular degradation of NLRP3 inflammasome components (13, 14). To investigate whether F240B affects the protein stability of NLRP3 and proIL-1β, J774A.1 macrophages were incubated with LPS for 5 h followed by incubation with F240B or DMSO (vehicle) for 3 h. The cells were then treated with the translation inhibitor cycloheximide (CHX) for 3-12 h. The protein expression levels of NLRP3 and proIL-1β were measured by Western blotting. We found that NLRP3 protein was not significantly degraded after 3-12 h CHX treatment in DMSO-treated cells; however, NLRP3 protein was significantly degraded after 12 h CHX treatment in F240B-treated cells (**Figure 6A**). In addition, proIL-1β protein was degraded in DMSO-treated cells in a time-dependent manner, and this effect was enhanced by F240B (**Figure 6A**). To investigate whether the autophagy-dependent pathway was involved in F240B-mediated degradation of NLRP3 and proIL-1β, the autophagy inhibitor 3-MA was used. We found a substantial accumulation of NLRP3 and proIL-1β protein levels upon treatment with 3-MA (**Figure 6B**). These results indicate that the autophagy-dependent pathway contributes to the degradation of NLRP3 and proIL-1β in F240B-treated cells.

### Activation of Autophagy by F240B Limits Mitochondrial Integrity Loss

Induction of proIL-1β and NLRP3 is not sufficient to activate the NLRP3 inflammasome. Fully activating the NLRP3 inflammasome requires an additional stimulus that provides activation signals (15). Mitochondrial dysfunction is an important activation signal for the NLRP3 inflammasome (16). We found that ATP induced mitochondrial membrane integrity loss (evidenced by reduced MitoTracker Deep Red staining; R2 fraction) in J774A.1 macrophages, and this effect was reduced by F240B (**Figure 7A**). Importantly, the autophagy inhibitor 3-MA limited the protective effect of F240B on mitochondria (**Figure 7A**). In addition, the protective effect of F240B on mitochondria was also reduced in LC3 knockout J774A.1 macrophages compared to that in wild-type cells (**Figure 7B**). These results indicate that activation of autophagy by F240B limits mitochondrial integrity loss and reduces NLRP3 inflammasome activation.

### F240B Inhibits ASC Oligomerization

Activation of the NLRP3 inflammasome is required the formation of high-molecular-weight ASC oligomers (17). To investigate whether F240B inhibits the NLRP3 inflammasome by reducing ASC oligomerization, the effect of F240B on ASC oligomerization was analysed by ASC speck formation and ASC cross-linking assays. We found that ATP induced ASC oligomerization, as evidenced by the formation of ASC specks in ASC-GFP-expressing J774A.1 macrophages, and this effect was reduced by F240B (**Figure 8A**). In addition, the inhibitory effect of F240B on ASC oligomerization was confirmed by ASC cross-linking Western blotting assays (**Figure 8B**). These results indicate that F240B inhibits ASC oligomerization. NLRP3 physically interacts with ASC, NEK7 and PKR, forming a protein complex that leads to NLRP3 inflammasome activation. Using immunoprecipitation and Western blotting assays, we found that the interactions between NLRP3 and ASC (**Figure 8C**) or NEK7 (**Figure 8D**) and PKR (**Figure 8E**) were not affected by F240B.

### F240B Inhibits NO, COX-2, and IL-6 Expression

To investigate whether F240B also inhibits the traditional inflammatory response independent of the NLRP3 inflammasome, the effect of F240B on the expression levels of NO, IL-6, TNF-α and COX-2 in LPS-activated J774A.1 macrophages was studied. We found that F240B inhibited LPS-induced COX-2 (**Figure 9A**), NO (**Figure 9B**) and IL-6 (**Figure 9C**) expression in a dose-dependent manner; however, LPS-induced TNF-α expression was not affected by F240B (**Figure 9D**). These results indicate that F240B not only inhibits the NLRP3 inflammasome but also reduces the traditional inflammatory response in macrophages.

### F240B Inhibits the NLRP3 Inflammasome and Inflammation in a Mouse Model of Gouty Inflammation

We investigated the *in vivo* anti-inflammatory activity of F240B using an NLRP3 inflammasome-associated MSU crystal-induced mouse peritonitis model (18). We found that intraperitoneal injection of MSU crystals increased neutrophil recruitment in the peritoneum, and this effect was significantly reduced by intraperitoneal injection of F240B (20 mg/kg body weight) and colchicine (1 mg/kg body weight) (**Figure 10A**). MSU crystal injection also increased the levels of IL-1β and active caspase-1 in the peritoneal lavage fluid, and these effects were reduced by F240B and colchicine (**Figure 10B**). In addition, F240B also reduced the levels of IL-6 and MCP-1 in the peritoneal lavage fluid of MSU crystal-injected mice (**Figure 10C**). These results indicate that F240B limits NLRP3 inflammasome activation and inflammation *in vivo*.

## Discussion

Autophagy is a catabolic cellular homeostatic process that is conserved across mammalian cell types. This self-destructive process is intended to ward off intracellular misfolded proteins and damaged organelles to maintain genome stability and energy balance in the cells. Autophagy is a cell survival mechanism that fights cellular stress, which otherwise results in cell death (19). Studies have shown that autophagy deficiency leads to extensive cellular stress and genome damage (20) and is a crucial bactericidal mechanism that drives bacteria-containing phagosomes to lysosomes for degradation (21). Thus autophagy plays important roles in the immune response. Earlier studies have shown that autophagy is controlled by a broad range of proteins encoded by autophagy-related genes, such as LC3, which is a common marker used for monitoring autophagosomes (12). Accumulating evidence has demonstrated that autophagy negatively regulates NLRP3 inflammasome activation (22). In this study, the autophagy-inducing ability of F240B was demonstrated by analyzing the autophagic degradation of p62, accumulation of LC3-II, LC3 speck formation and formation of acidic vesicular organelles (**Figure 1**). Autophagy inhibits the NLRP3 inflammasome by removing damaged mitochondria, which produces activation signals, including mitochondrial ROS and oxidized mitochondrial DNA (16, 23). We found that F240B inhibited the NLRP3 inflammasome by preserving mitochondrial integrity in an autophagy-dependent manner (**Figure 7**). Autophagy also inhibits the NLRP3 inflammasome by promoting the degradation of the inflammasome components NLRP3 and proIL-1β (13, 14). Although F240B did not inhibit NLRP3 expression, it significantly inhibited proIL-1β expression (**Figure 5A**). Notably, F240B-mediated proIL-1β downregulation was reversed by LC3 knockout (**Figure 5F**), indicating that autophagy promotes the degradation of proIL-1β in F240B-treated cells. It has been demonstrated that a low level of ROS positive regulate the priming signal of the NLRP3 inflammasome and promote NLRP3 inflammasome activation; however, Erttmann and Gekara demonstrated that a high level of ROS release by *Streptococcus pneumonia* increased the oxidative levels of the inflammasome components ASC and caspases and inhibited the NLRP3 inflammasome (24). These results may partially explain why F240B inhibited the NLRP3 inflammasome, but increased ROS production in LPS-activated macrophages (**Figure 5D**).

In the NLRP3 inflammasome, the adaptor protein ASC plays a key role in NLRP3 inflammasome assembly by interacting with NLRP3 *via* an N-terminal pyrin domain and recruiting procaspase-1 *via* a C-terminal caspase recruitment domain (25). Upon activation, ASC bridges NLRP3 and caspase-1 to form ternary inflammasome complexes, and oligomerization of ASC into filaments and the formation of an ASC speck is a critical step in NLRP3 inflammasome activation (17, 26). Thus, disruption of inflammasome complex assembly by targeting ASC is a novel strategy to inhibit the NLRP3 inflammasome (27). In our current study, we demonstrated that F240B inhibited ASC oligomerization and speck formation; however, F240B did not affect the interaction between ASC and NLRP3 (**Figure 8**). These results suggest that F240B binds to the amino acid residues in ASC, which is critical for ASC polymerization and fibril extension, but does not bind to the site that recruits NLRP3 (28). In addition, two NLRP3 binding proteins, NEK7 and PKR, have been shown to positively regulate the activation of the NLRP3 inflammasome (29, 30). NEK7 acts downstream of potassium efflux to specifically regulate NLRP3 inflammasome activation, as NEK7 knockout results in reduced caspase-1 activation and IL-1β release in NLRP3-activated macrophages but not in NLRC4- or AIM2-activated macrophages (29). Unlike NEK7, PKR broadly regulates inflammasome activation by physically interacting with NLRP3, NLRP1, NLRC4 and AIM2 (30). However, in this study, we found that the NLRP3-NEK7 and NLRP3-PKR interactions were unaltered by the presence of F240B (**Figure 8**). The molecular mechanism by which F240B interrupts ASC oligomerization is a wide platform that remains to be investigated.

Over the past decade, NLRP3 inﬂammasome has become a promising molecular target in the fight against inflammatory diseases (1). Research efforts have focused on understanding the pathogenic roles of the NLRP3 inflammasome in metabolic syndromes, cardiovascular diseases and neurologic disorders (31). In our current study we demonstrated that F240B inhibits NLRP3 inflammasome in macrophages. Although we demonstrated that F240B ameliorates MSU crystal-induced peritonitis in a mouse model (**Figure 10**), the limitation of this study is the lack of pathophysiological relevant evaluation for F240B. It is better to confirm the *in vivo* anti-inflammatory activity of F240B using NLRP3-associated disease models, such as subcutaneous air pouch inflammation model (27) or gout inflammation model by injecting MSU crystals in joints (32). Another imitation of this study is only using single dose of F240B to evaluate the anti-inflammatory *in vivo*. Although F240B (IC_50_ < 1 µM) is more effective to inhibit NLRP3 inflammasome than its analogue 4-HAB (IC_50_ ~20 µM) in macrophages, without comparison of the *in vivo* dose-dependent inhibition of F240B we cannot conclude that F240B has more effective anti-inflammatory effects than 4-HAB, and more detailed investigation is need.

In summary, we have synthesized a new polyenylpyrrole derivative F240B and demonstrated that it is a potential anti-inflammatory agent that inhibits the activation of NLRP3 inflammasome by inducing autophagy in macrophages (**Figure 11**). We further demonstrated that F240B exerts *in vivo* anti-inflammatory activity in a mouse model of uric acid crystal-induced peritonitis, which is an NLRP3 inflammasome-associated disorder (33). As abnormal activation of the NLRP3 inflammasome leads to a broad range of inflammatory disorders, F240B has the potential to ameliorate NLRP3-associated diseases, including chronic kidney disease (34–36), inflammatory bowel disease (37) and neurodegenerative disorders (38). However, to assess the therapeutic potential of F240B, *in vitro* safety pharmacology studies and the *in vitro* and *in vivo* pharmacokinetics of F240B should be addressed.

## Data Availability Statement

The original contributions presented in the study are included in the article/**Supplementary Material**; further inquiries can be directed to the corresponding authors.

## Ethics Statement

The animal study was reviewed and approved by Institutional Animal Care and Use Committee of the National Ilan University.

## Author Contributions

K-FH is the guarantor of the article. C-HW, YL, and K-FH conceived and designed the study. C-HW, CG, L-HL, J-CC, and S-TC performed the experiments and analyzed the data. C-LH and OC assisted with some experiments. MM, S-MC, S-PY, and C-HL contributed to critical revision of the manuscript. C-HW, YL, and K-FH wrote and finished the manuscript. All authors contributed to the article and approved the submitted version.

## Funding

This research work is supported by the funding from the Ministry of Science and Technology of Taiwan (MOST 109-2628-B-197-001, MOST 109-2321-B-197-006, MOST 109-2811-B-197-500, MOST 108-2923-B-197-001-MY3) and Tri-Service General Hospital, Taipei, Taiwan (TSGH-C108-024, TSGH-D-109039).

## Acknowledgments

We thank Dr. Wei-Ting Wong at National Ilan University for her critical revision.

## Supplementary Material

The Supplementary Material for this article can be found online at: https://www.frontiersin.org/articles/10.3389/fimmu.2020.607564/full#supplementary-material

## Conflict of Interest

The authors declare that the research was conducted in the absence of any commercial or financial relationships that could be construed as a potential conflict of interest.
