# Corrigendum: A Synthetic Small Molecule F240B Decreases NLRP3 Inflammasome Activation by Autophagy Induction

**DOI:** 10.3389/fimmu.2021.738591

**Published:** 2021-12-15

**Authors:** Chun-Hsien Wu, Chin Heng Gan, Lan-Hui Li, Jen-Che Chang, Shin-Tai Chen, Mridula P. Menon, Shu-Meng Cheng, Shih-Ping Yang, Chen-Lung Ho, Oleg V. Chernikov, Chi-Hung Lin, Yulin Lam, Kuo-Feng Hua

**Affiliations:** ^1^ Division of Cardiology, Department of Internal Medicine, Tri-Service General Hospital, National Defense Medical Center, Taipei, Taiwan; ^2^ Institute of Microbiology and Immunology, National Yang-Ming University, Taipei, Taiwan; ^3^ Department of Chemistry, National University of Singapore, Singapore, Singapore; ^4^ Department of Laboratory Medicine, Linsen, Chinese Medicine and Kunming Branch, Taipei City Hospital, Taipei, Taiwan; ^5^ Department of Pathology, Tri-Service General Hospital, National Defense Medical Center, Taipei, Taiwan; ^6^ Department of Biotechnology and Animal Science, National Ilan University, Ilan, Taiwan; ^7^ Division of Wood Cellulose, Taiwan Forestry Research Institute, Taipei, Taiwan; ^8^ G.B. Elyakov Pacific Institute of Bioorganic Chemistry FEB RAS, Vladivostok, Russia; ^9^ Department of Biological Science & Technology, National Chiao Tung University, Hsinchu, Taiwan; ^10^ Department of Medical Research, China Medical University Hospital, China Medical University, Taichung, Taiwan

**Keywords:** NLRP3 inflammasome, conjugated polyenes, autophagy, mitochondria, peritonitis

In the original article, there was a mistake in **Figures 5E** and **8E** as published. We found a mismatch between the Western blot images (p-JNK1/2) in comparison to the labeling in **Figures 5E**. Regarding the effect of F240B on LPS-induced phosphorylation of ERK1/2, p38 and JNK1/2, we tested two time-course studies: (A) condition was LPS treatment for 0, 10, 20, 30 min (total eight groups), (A) condition was LPS treatment for 0, 10, 20, 30, 60 min (total ten groups). In the **Figures 5E** of the original manuscript, we used (A) condition for ERK1/2, p38; however, we used (B) condition for JNK. In addition, in **Figures 8E** of the original manuscript, the Western blot image of input NLRP3 included a non-specific band (far left band), making it looks like a mismatch between input NLRP3 and input PKR. The corrected **Figures 5E** and **8E** appear below.

**Figure 5:**
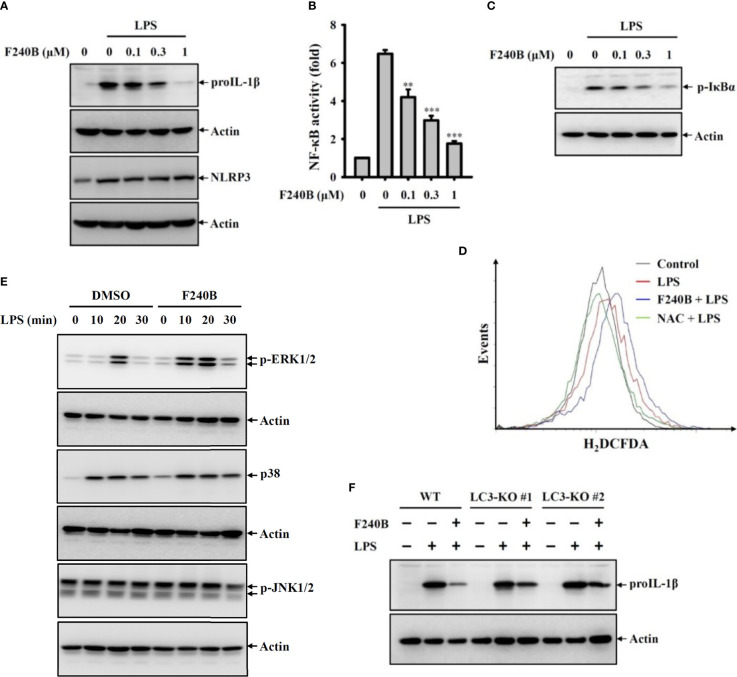
Activation of autophagy by F240B inhibits NF-kB activation and proIL-1b expression. **(A)** J774A.1 macrophages were incubated with F240B for 0.5 h followed by incubated with 1 μg/ml LPS for 6 h. The levels of proIL-1b and NLRP3 in the cell lysates were measured by Western blotting. **(B)** J-Blue cells were incubated with F240B for 0.5 h followed by incubated with 1 μg/ml LPS for 24 h. The NF-kB transcriptional activity was measured by NF-kB reporter assay. **(C, D)** J774A.1 macrophages were incubated with F240B (1 μM for ROS assay) for 0.5 h followed by incubated with 1 μg/ml LPS for 10 min. The phosphorylation levels of IkBa in the cell lysates were measured by Western blotting **(C)**, and the intracellular ROS production was analysed by H2DCFDA staining **(D)**. **(E)** J774A.1 macrophages were incubated with 1 μM F240B for 0.5 h followed by incubated with 1 μg/ml LPS for 10-30 min. The phosphorylation levels of ERK1/2, JNK1/2 and p38 in the cell lysates were measured by Western blotting. **(F)** Will-type or LC3-knockout J774A.1 macrophages were incubated with1 μM F240B for 0.5 h followed by incubated with 1 μg/ml LPS for 6 h. The levels of proIL-1b in the cell lysates were measured by Western blotting. ** and *** indicate a significant difference at the level of *p* < 0.01 and *p* < 0.001, respectively compared to LPS.

**Figure 8:**
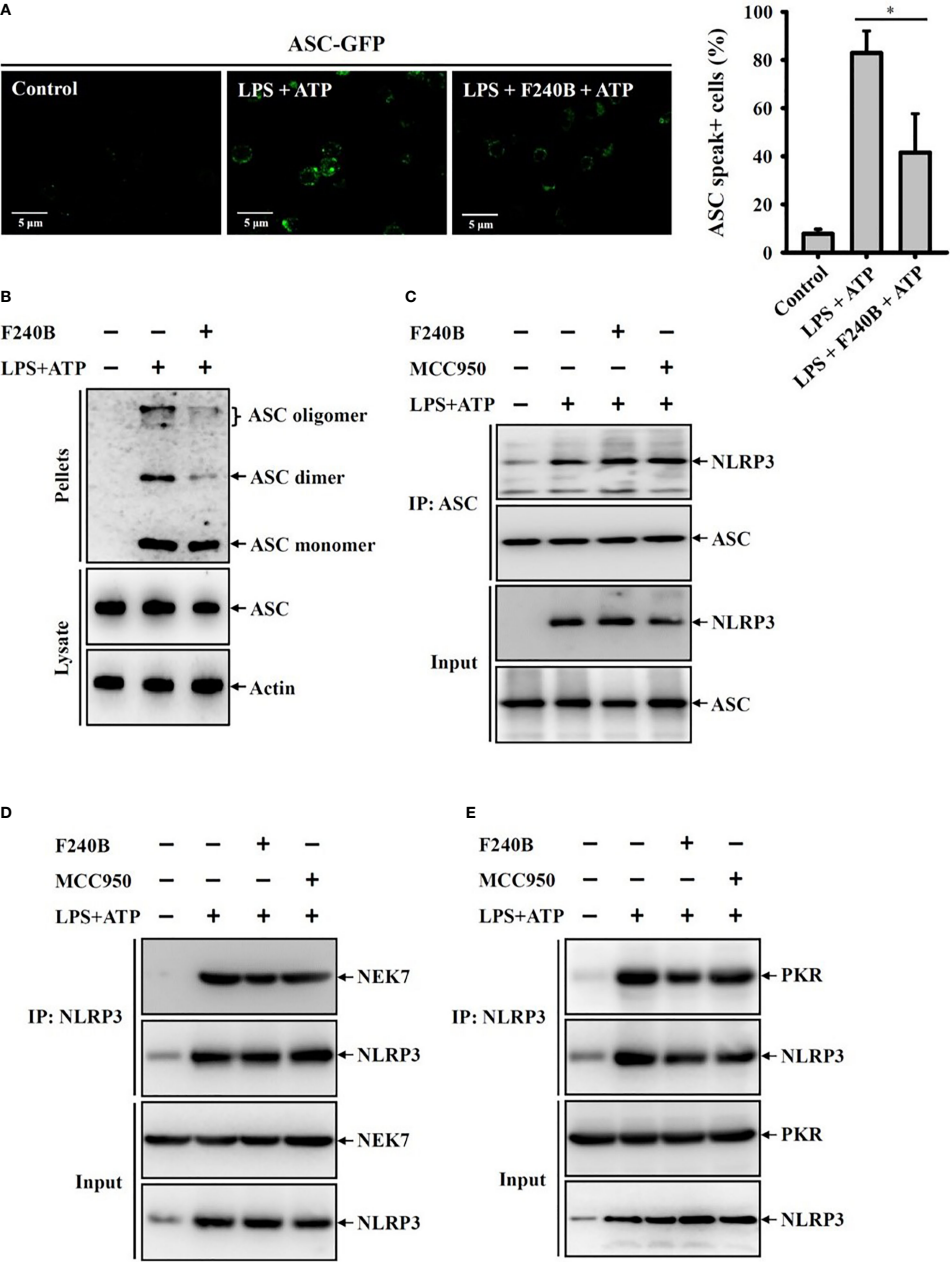
F240B inhibits ASC oligomerization. **(A)** ASC-GFP expressed J774A.1 macrophages or **(B)** J774A.1 macrophages were incubated with 1 μg/ml LPS for 5 h followed by incubated with 1 μM F240B for 3 h. Cells then incubated with 5 mM ATP for 0.5 h. The ASC speck formation was analyzed by fluorescent microscope **(A)**, or the cell lysates were crosslinked by disuccinimidyl suberate and ASC oligomerization was analyzed by Western blotting **(B)**. **(C–E)** J774A.1 macrophages were incubated with 1 μg/ml LPS for 5 h followed by incubated with 1 μM F240B or 0.1 μM MCC950 for 3 h. Cells then incubated with 5 mM ATP for 0.5 h. The interaction between NLRP3 with ASC **(C)**, NEK7 **(D)** or PKR **(E)** were analyzed by immunoprecipitation and Western blotting assay. The percentage of ASC speck positive cells are expressed as the mean ± SD of three separate experiments. * indicates a significant difference at the level of *p* < 0.05.

The authors apologize for this error and state that this does not change the scientific conclusions of the article in any way. The original article has been updated.

## Publisher’s Note

All claims expressed in this article are solely those of the authors and do not necessarily represent those of their affiliated organizations, or those of the publisher, the editors and the reviewers. Any product that may be evaluated in this article, or claim that may be made by its manufacturer, is not guaranteed or endorsed by the publisher.

