# Supplementary material for: A Synthetic Small Molecule F240B Decreases NLRP3 Inflammasome Activation by Autophagy Induction
Source: Front Immunol. 2020 Dec 18;11:607564. doi: 10.3389/fimmu.2020.607564 (PMC7793731; doi:10.3389/fimmu.2020.607564)
Supplement: Supplementary file 1 [file DataSheet_1.pdf]

Supporting Information

A synthetic small molecule F240B decreases NLRP3 inflammasome activation by autophagy induction

Chun-Hsien Wu[1,2], Chin Heng Gan[3], Lan-Hui Li[4,5], Jen-Che Chang[6], Shin-Tai Chen[6], Mridula P. Menon[6], Shu-Meng Cheng[1], Shih-Ping Yang[1], Chen-Lung Ho[7], Oleg V. Chernikov[8], Chi-Hung Lin[2,9], Yulin Lam[3,*], Kuo-Feng Hua[5,6,10,*]

[1]Division of Cardiology, Department of Internal Medicine, Tri-Service General Hospital, National Defense Medical Center, Taipei, Taiwan

[2]Institute of Microbiology and Immunology, National Yang-Ming University, Taipei, Taiwan

[3]Department of Chemistry, National University of Singapore, 3 Science Drive 3, Singapore 117543

[4]Department of Laboratory Medicine, Linsen, Chinese Medicine and Kunming Branch, Taipei City Hospital, Taipei, Taiwan

[5]Department of Pathology, Tri-Service General Hospital, National Defense Medical Center, Taipei, Taiwan

[6]Department of Biotechnology and Animal Science, National Ilan University, Ilan, Taiwan

[7]Division of Wood Cellulose, Taiwan Forestry Research Institute, Taipei, Taiwan

[8]G.B. Elyakov Pacific Institute of Bioorganic Chemistry FEB RAS, Vladivostok, Russia

[9]Department of Biological Science & Technology, National Chiao Tung University, Hsinchu, Taiwan

[10]Department of Medical Research, China Medical University Hospital, China Medical University, Taichung, Taiwan

*Correspondence: chmlamyl@nus.edu.sg; Tel.: +65-6516-2688 (Y Lam);
kuofenghua@gmail.com; Tel.: +886-3931-7630 (KF Hua).

Figure 1

^{1}H NMR spectrum of 3

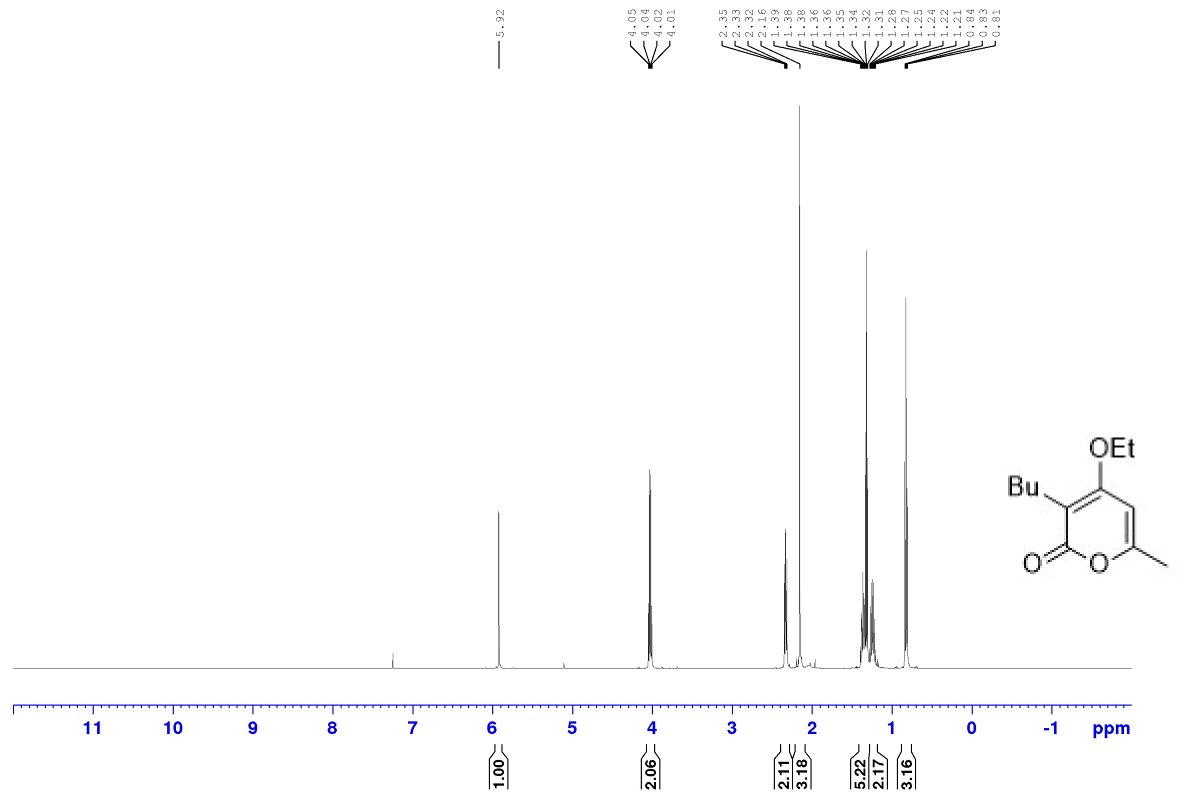

Figure 2

^{13}C NMR spectrum of 3

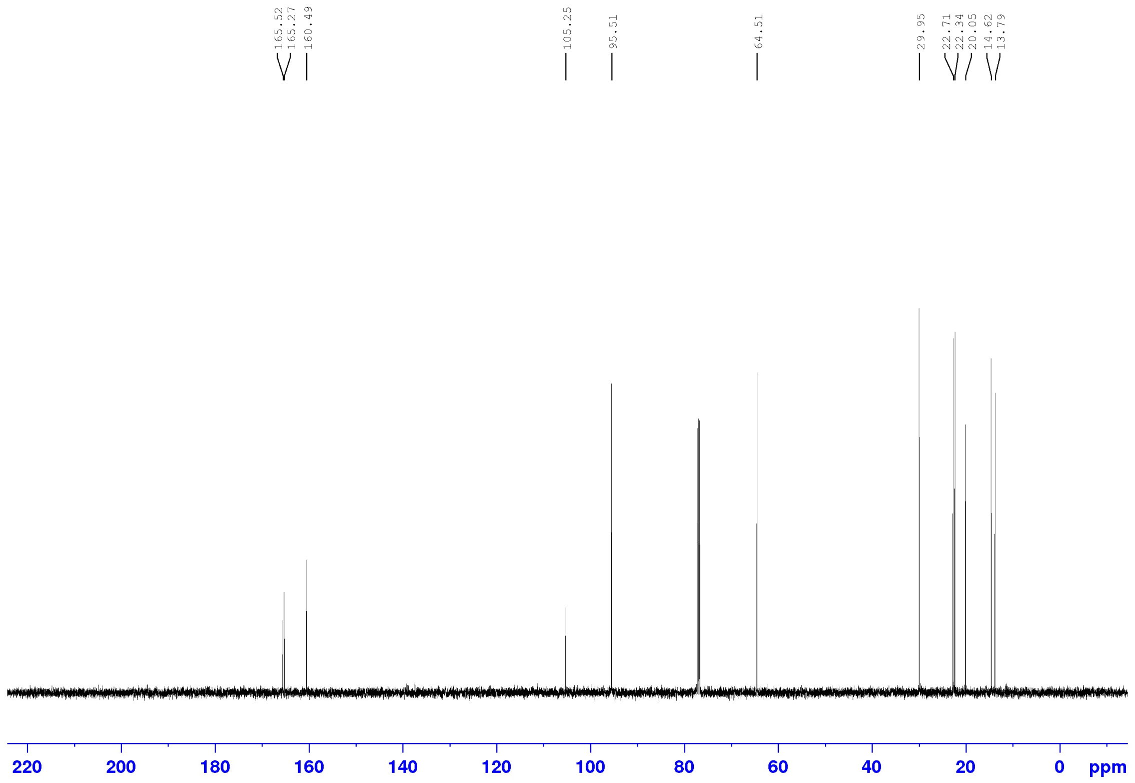

Figure 3

^{1}H NMR spectrum of 4

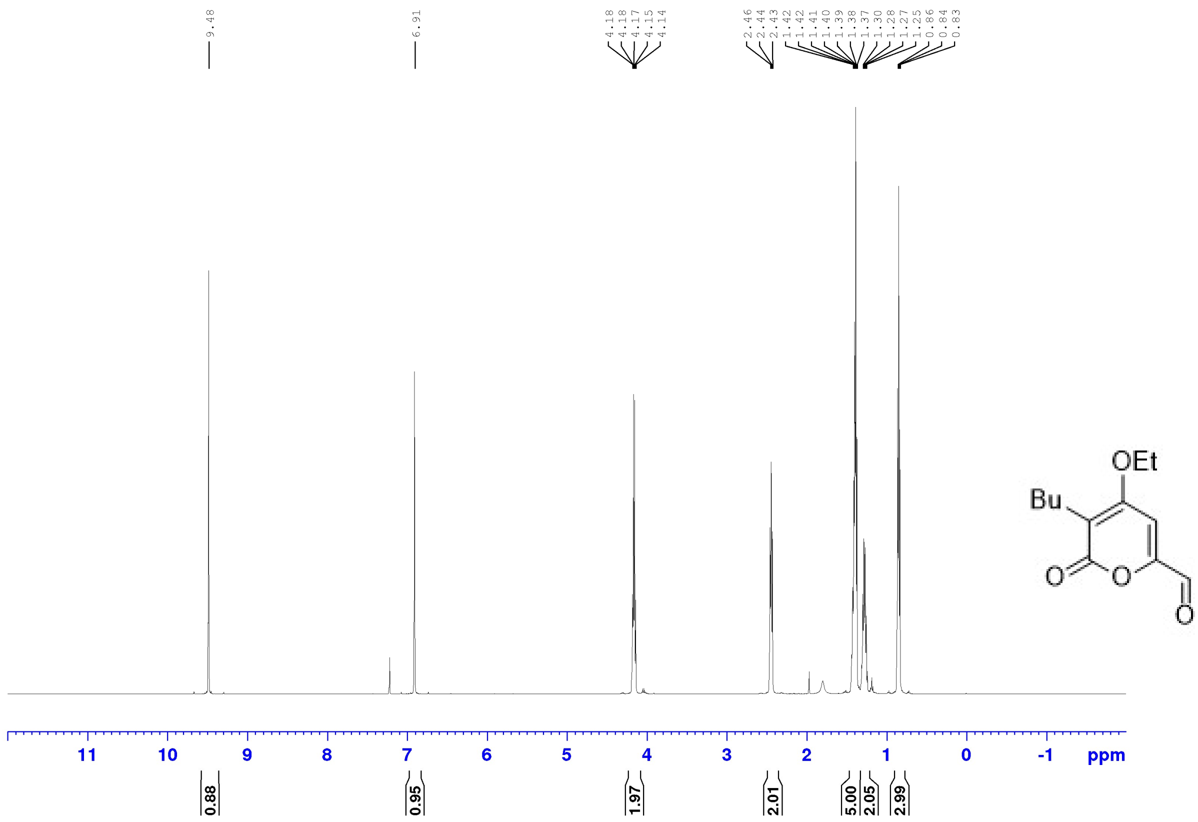

Figure 4

^{13}C NMR spectrum of 4

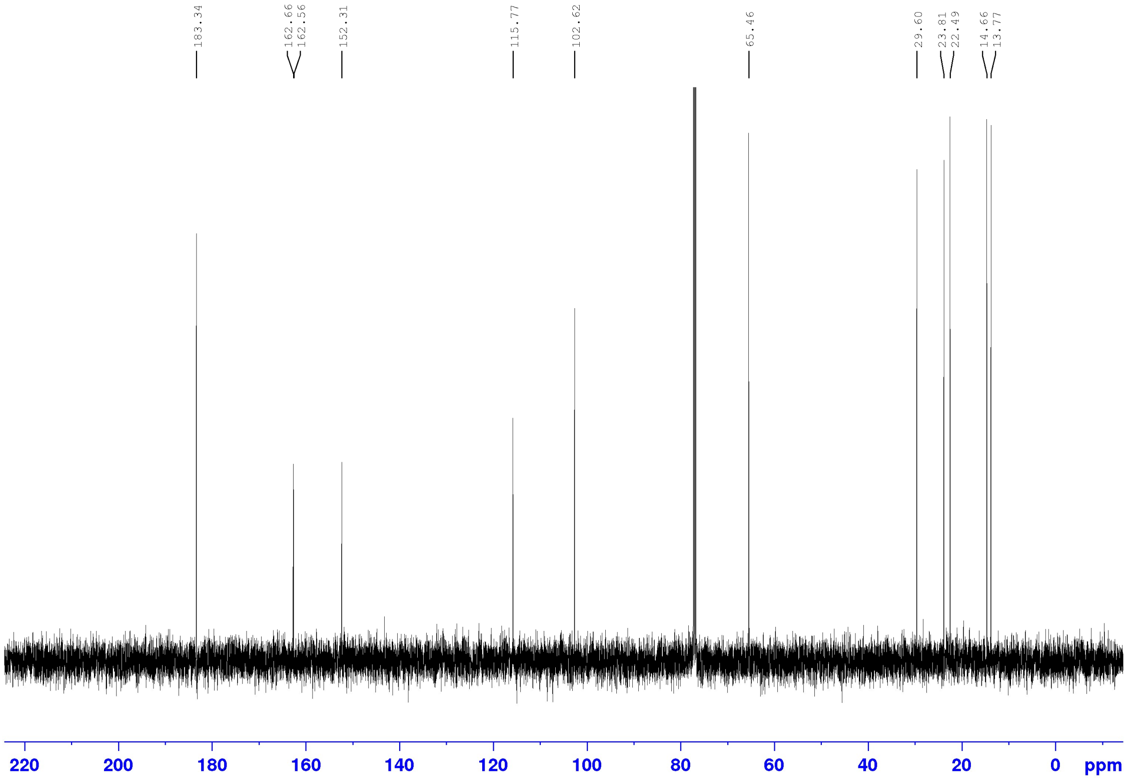

Figure 5

^{1}H NMR spectrum of 6

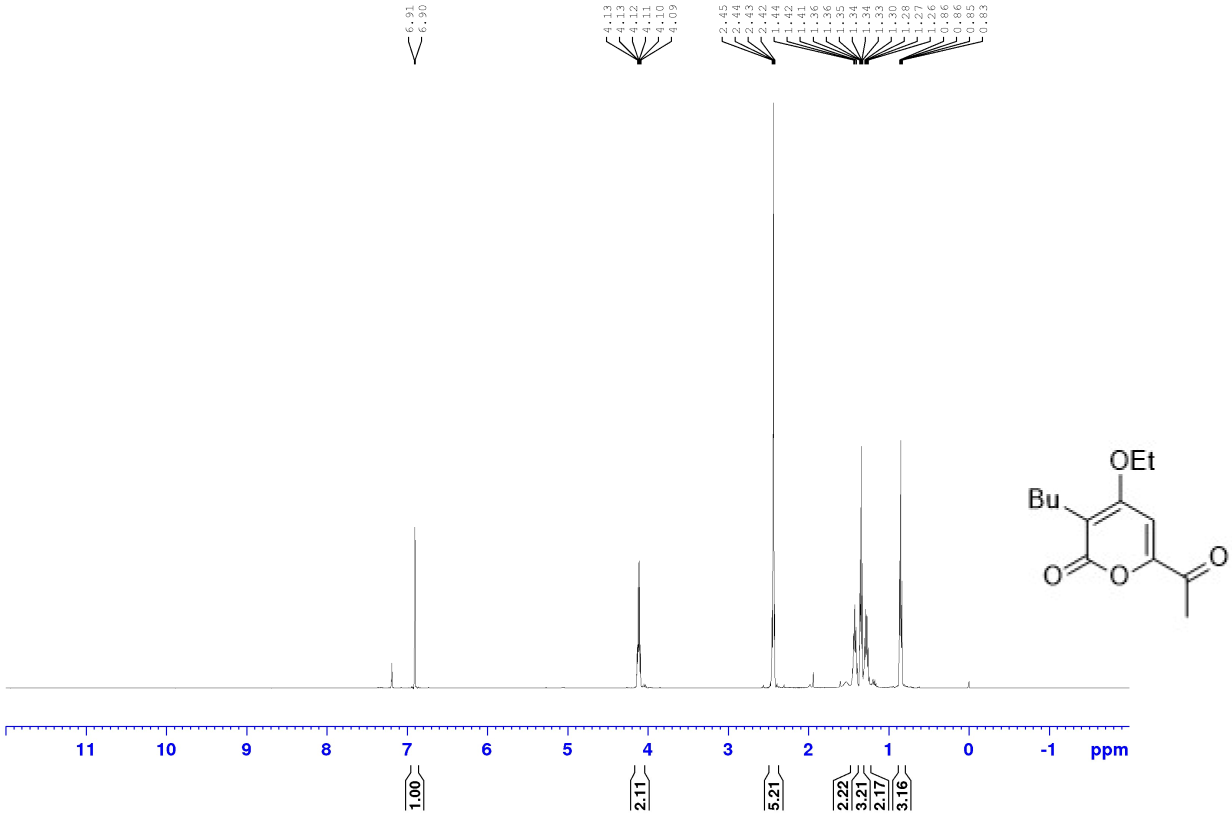

Figure 6

^{13}C NMR spectrum of 6

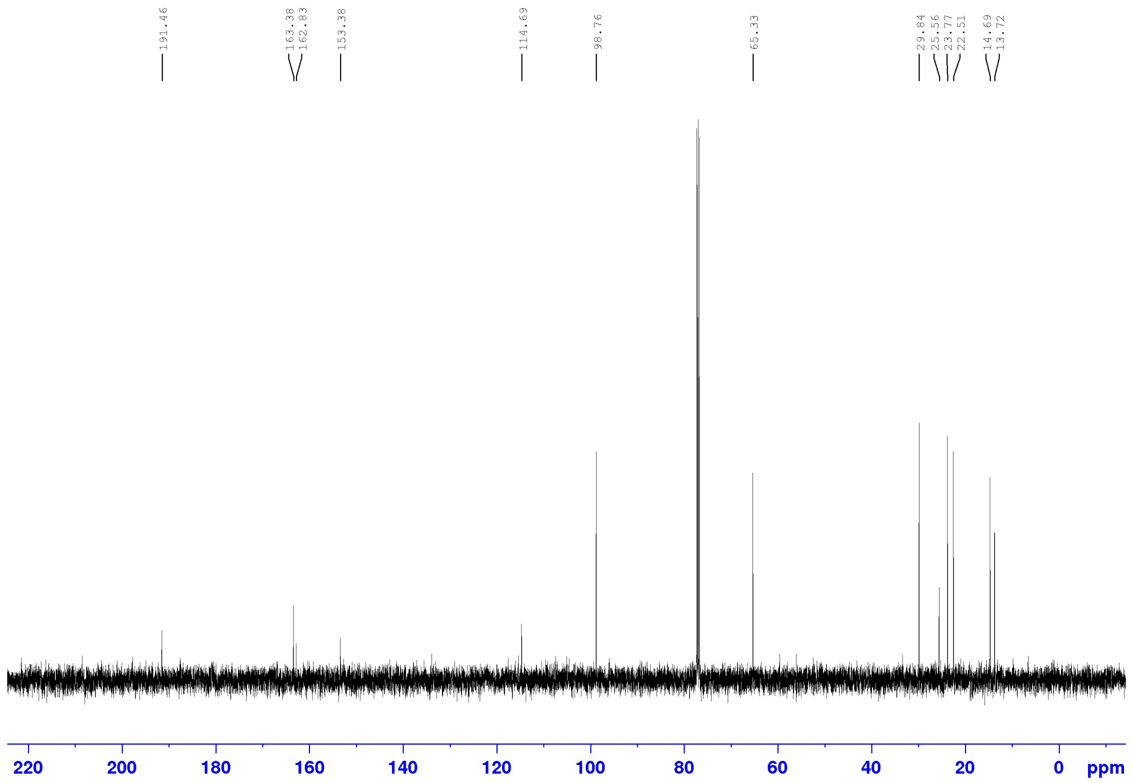

Figure 7

^{1}H NMR spectrum of 7

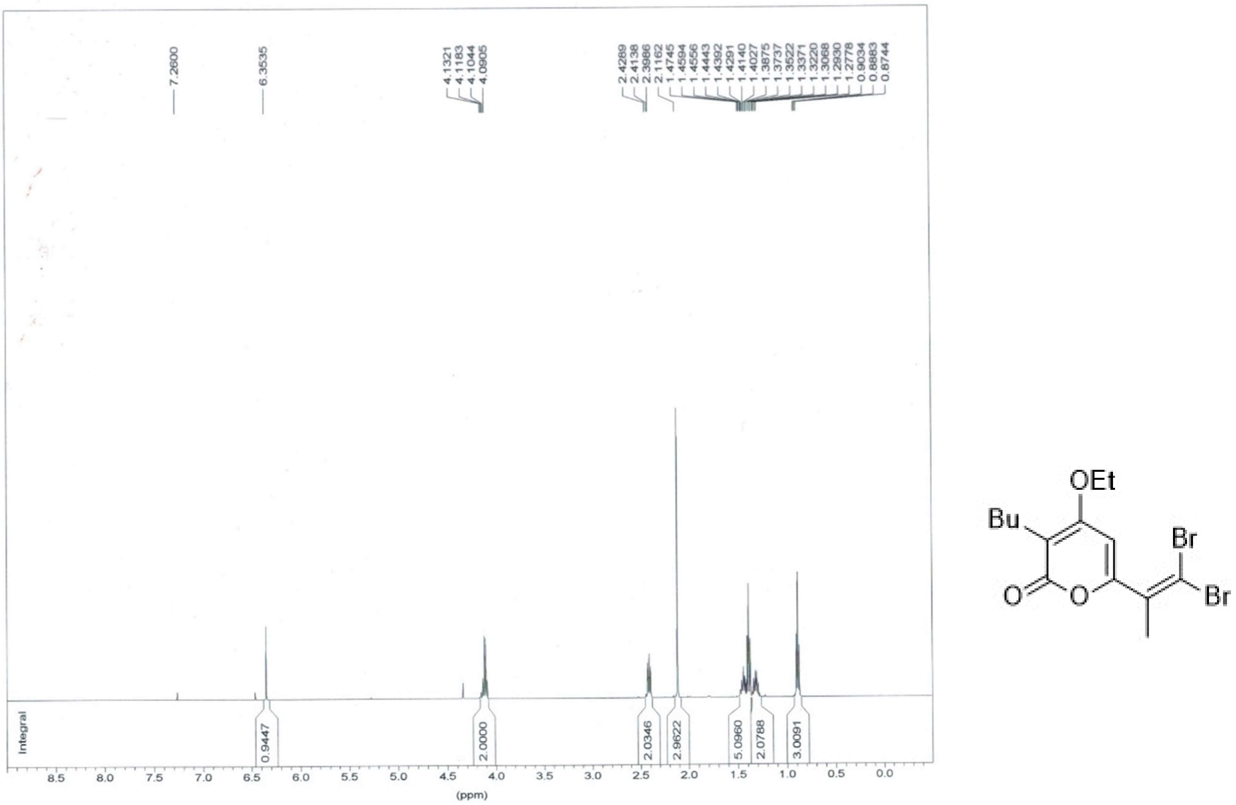

Figure 8

^{13}C NMR spectrum of 7

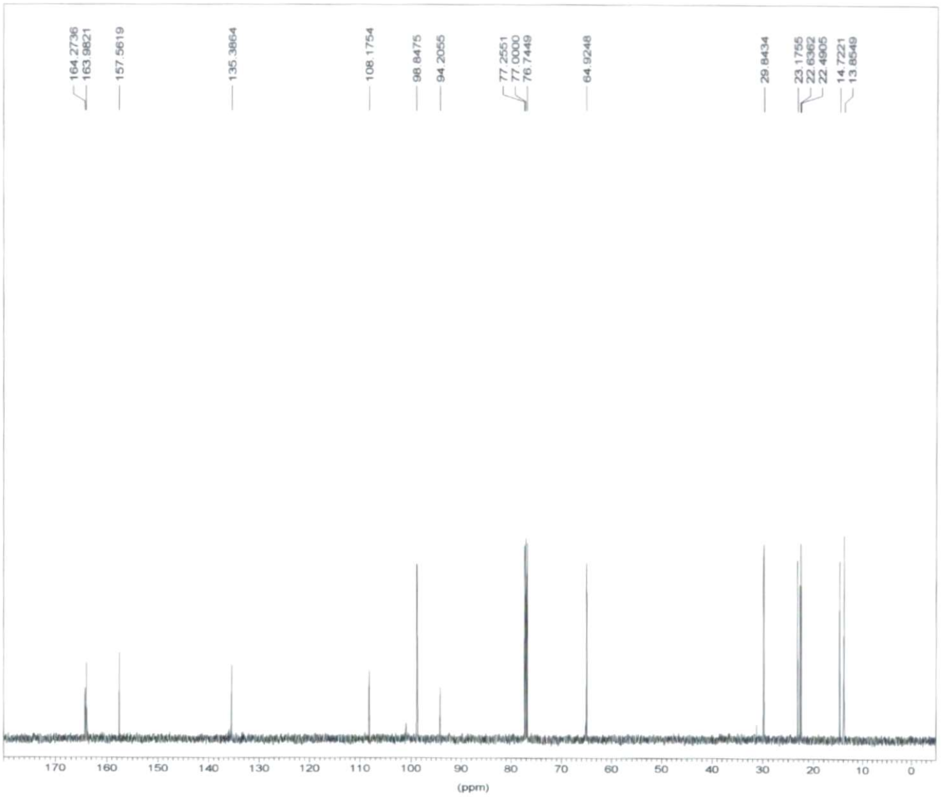

Figure 9

^{1}H NMR spectrum of 1

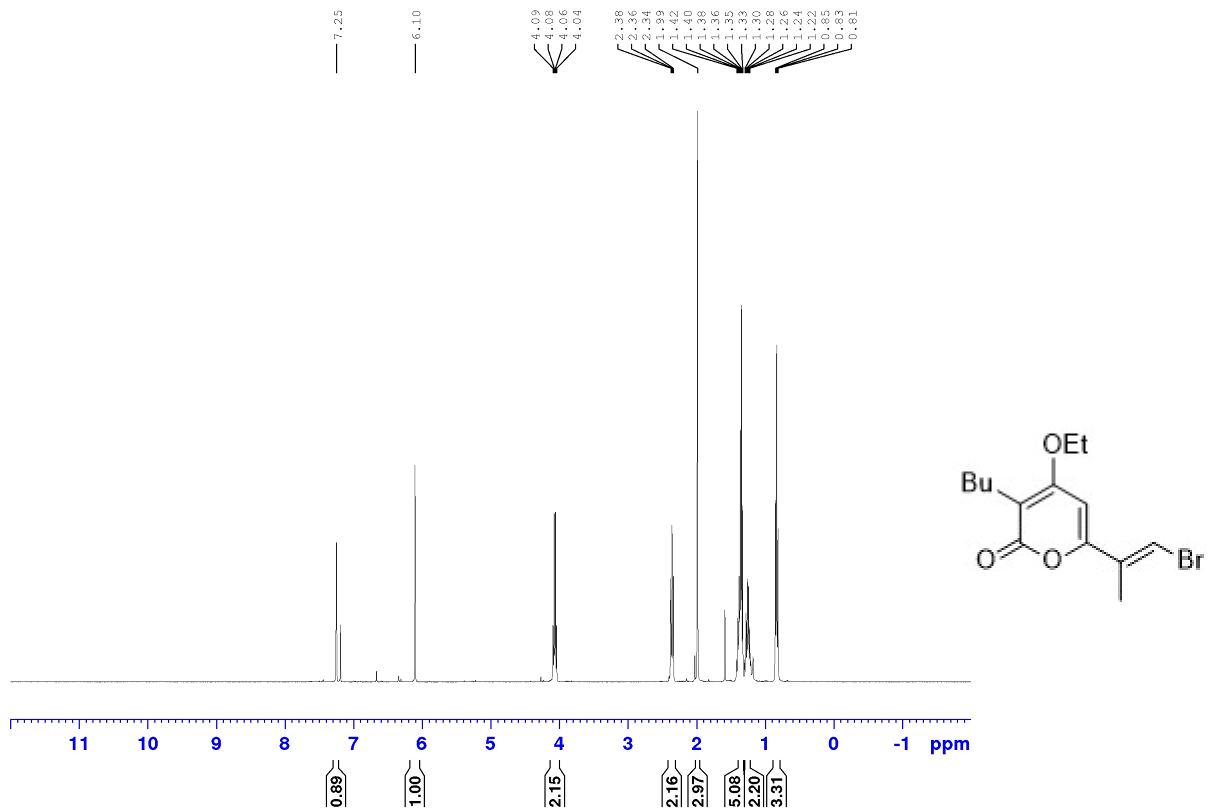

Figure 10

^{13}C NMR spectrum of 1

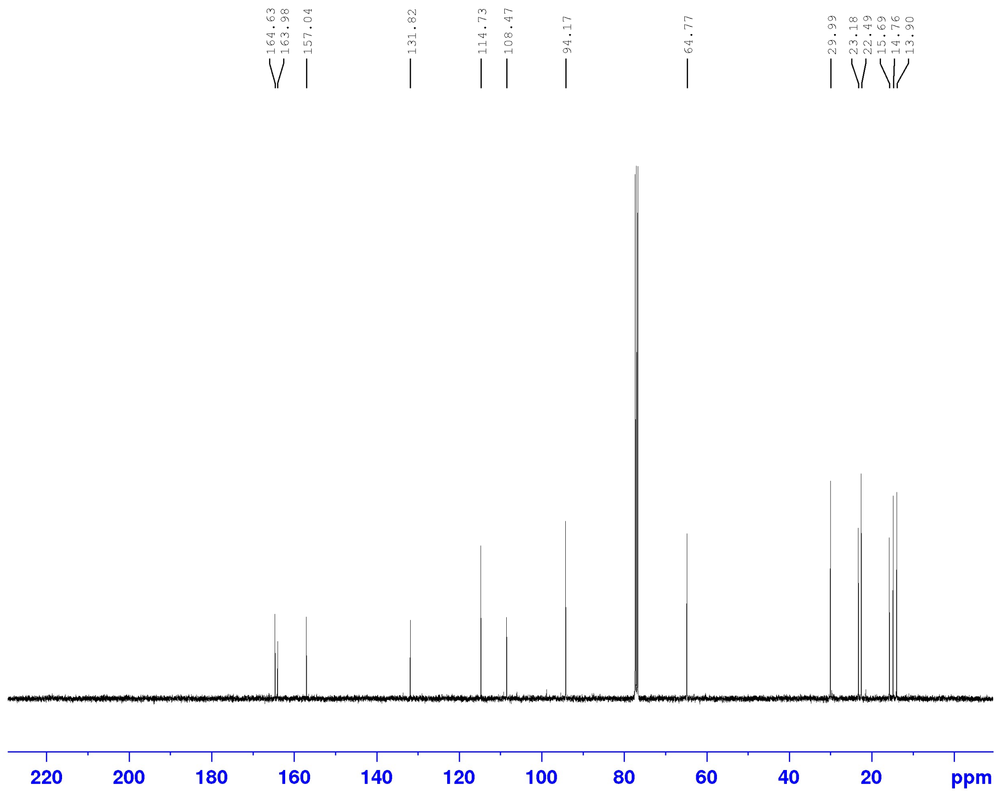

Figure 11

^{1}H NMR spectrum of F240B

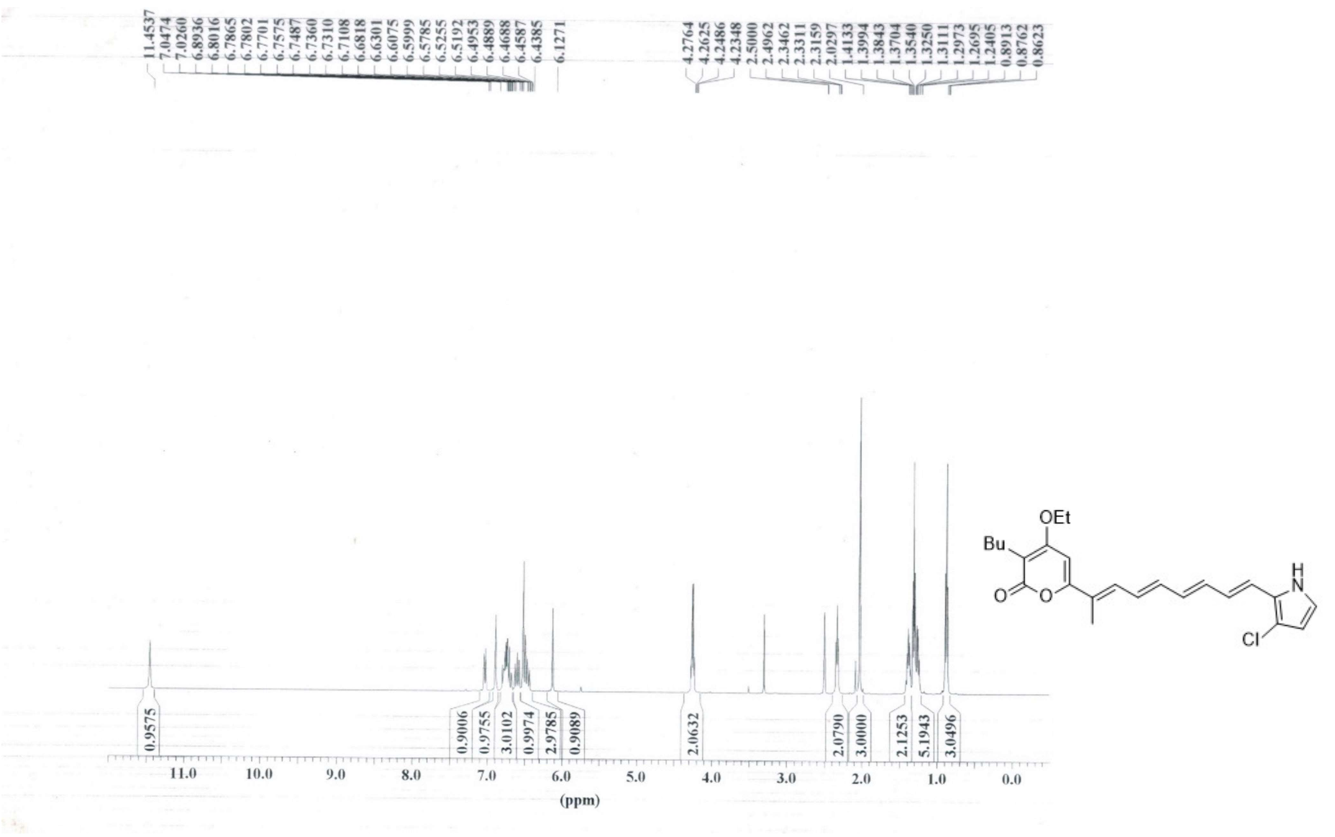

Figure 12

^{13}C NMR spectrum of F240B

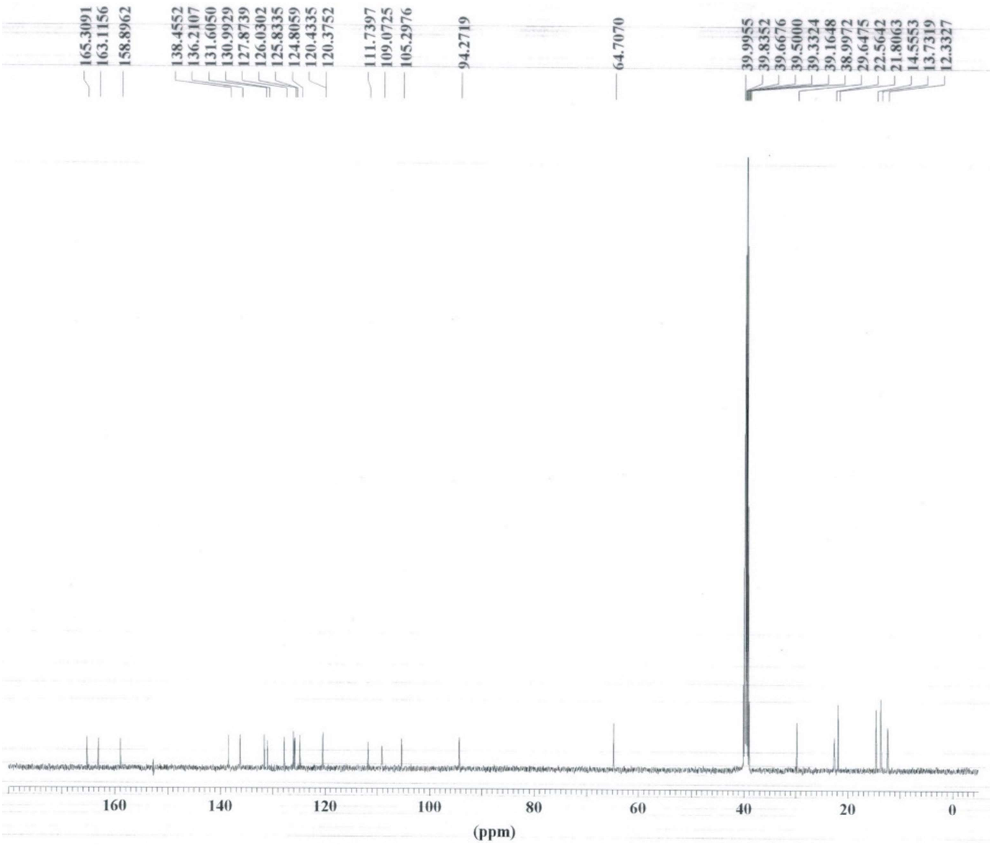
165.3091
163.1156
158.8962
138.4552
136.2107
131.6050
130.9929
127.8739
126.0302
125.8335
124.8059
120.4335
120.3752
111.7397
109.0725
105.2976
94.2719
64.7070
39.9955
39.8352
39.6676
39.5000
39.3324
39.1648
38.9972
29.6475
22.5642
21.8063
14.5553
13.7319
12.3327
160
140
120
100
80
60
40
20
0
(ppm)
